# Recent developments in sunscreens based on chromophore compounds and nanoparticles

**DOI:** 10.1039/d3ra08178h

**Published:** 2024-01-15

**Authors:** Mani Rajasekar, Jennita Mary, Meenambigai Sivakumar, Masilamani Selvam

**Affiliations:** a Centre for Molecular and Nanomedical Sciences, International Research Centre, Sathyabama Institute of Science and Technology (Deemed to be University) Chennai – 600 119 Tamil Nadu India mrajasekar_83@yahoo.com drmrajasekar.irc@sathyabama.ac.in +91-9710230530; b School of Bio and Chemical Engineering, Department of Biotechnology, Sathyabama Institute of Science and Technology (Deemed to be University) Chennai 600119 Tamil Nadu India

## Abstract

Sunscreen formulations have undergone significant advancements in recent years, with a focus on improving UV radiation protection, photostability, and environmental sustainability. Chromophore compounds and nanoparticles have emerged as key components in these developments. This review highlights the latest research and innovations in chromophore compounds and nanoparticle-based sunscreens. It discusses the role of nanoparticles, such as zinc oxide and titanium dioxide, in scattering and absorbing UV radiation while remaining cosmetically acceptable. Chromophore compounds, encapsulated in nanoparticles, are explored for their potential to enhance UV protection by absorbing specific wavelengths of light. Additionally, advances in photo-stability, broad-spectrum protection, antioxidant inclusion, and biodegradability are discussed. The evolving landscape of sunscreen technology aims to provide more effective and environment-friendly solutions for safeguarding skin from the sun's harmful effects.

## Abstract 2

Sunscreen formulations have undergone significant advancements in recent years, with a focus on improving UV radiation protection, photostability, and environmental sustainability.

## Graphical abstract

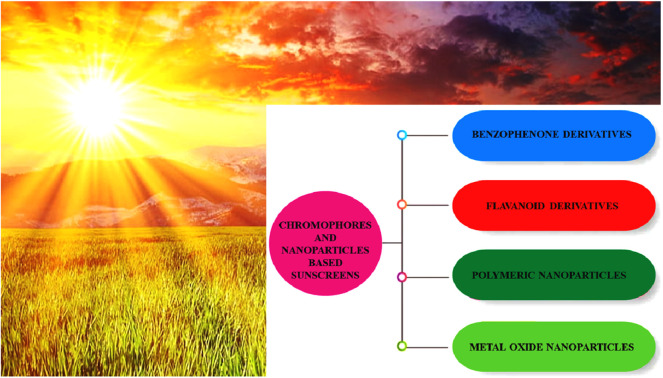

## 1. Introduction

Sunscreen is a vital product that helps protect our skin from the harmful effects of the sun's ultraviolet (UV) rays. It is a topical product that comes in various forms, such as lotions, creams, gels, sprays, and sticks.^1^ The primary purpose of sunscreen is to shield our skin from both UVA and UVB rays, which can cause sunburn and premature aging, and increase the risk of skin cancer. UVA rays penetrate deep into the skin and contribute to skin aging, while UVB rays primarily affect the outer layers of the skin and are the main cause of sunburn. Sunscreen works by either absorbing or reflecting these harmful rays, preventing them from damaging the skin. The effectiveness of sunscreen is measured by its sun protection factor (SPF), which indicates the level of protection it offers against UVB rays. The higher the SPF, the greater the protection.^2^ The difference in protection between SPF 30, 40, and 50 is relatively small, and no sunscreen can block 100% of UVB rays. It is recommended to use a broad-spectrum sunscreen with an SPF of 30 or higher to ensure adequate protection against both UVA and UVB rays. Applying sunscreen correctly is crucial for its effectiveness. It should be generously applied to all exposed areas of the skin at least 15 minutes before sun exposure. Reapplication is necessary every two hours, or more frequently if sweating or swimming, to maintain its protective effect. In addition to sunscreen, it is important to take other sun protection measures, such as seeking shade during peak sun hours, wearing protective clothing, and using sunglasses to shield the eyes from UV rays.^3^

**Fig. 1:**
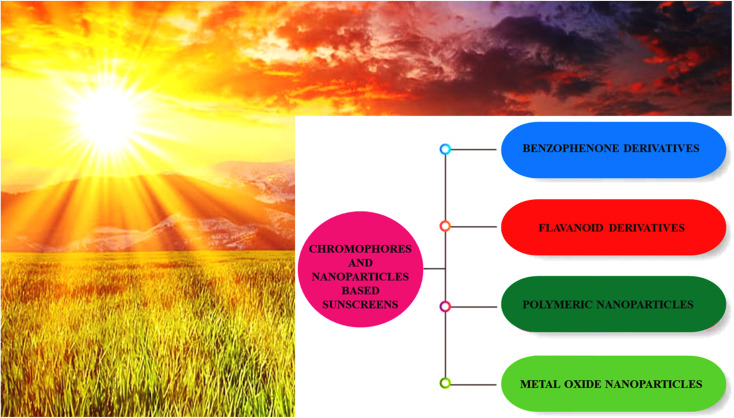
Schematic representation of chromophore compounds and nanoparticles-based sunscreens.

In addition, the chromophore-based sunscreen is an innovative approach to sun protection that utilizes specific molecules known as chromophores to absorb and dissipate UV radiation.^4^ These chromophores are designed to selectively absorb UV light, providing effective protection against both UVA and UVB rays. Unlike traditional sunscreens that rely on physical or chemical filters to block or scatter UV rays, chromophore-based sunscreens work by absorbing the UV radiation and converting it into less harmful forms of energy, such as heat.^5,6^ This mechanism allows for efficient and targeted protection against the damaging effects of the sun. It used in these sunscreens are carefully selected to have high absorption capabilities within the UV spectrum.^7–10^ They are designed to absorb specific wavelengths of UV light, ensuring broad-spectrum protection. By absorbing UV radiation, chromophores prevent it from penetrating the skin and causing damage, such as sunburn, premature aging, and an increased risk of skin cancer. They have the potential for improved photostability, meaning they are less likely to degrade or lose their effectiveness when exposed to sunlight. This ensures that the sunscreen remains active for a longer duration, providing reliable protection throughout sun exposure. It is important to note that extensive research is conducted to ensure the safety and efficacy of chromophore-based sunscreens. Regulatory bodies closely monitor these products to ensure they meet stringent standards for consumer safety (Fig. 1).^11–13^

Moreover, Nanoparticles-based sunscreen is a revolutionary advancement in sun protection technology. These sunscreens utilize tiny particles, typically ranging from 1 to 100 nanometers in size, to provide enhanced protection against the sun's harmful UV rays.^14^ The nanoparticles used in these sunscreens are often made of materials like titanium dioxide or zinc oxide. These materials can absorb, scatter, and reflect UV radiation, making them highly effective in shielding the skin from both UVA and UVB rays.^15–17^ Titanium dioxide and zinc oxide are commonly used in sunscreen formulations because they are effective at blocking both UVA and UVB rays, they are non-irritating to the skin, and they are considered to be non-toxic. Other metal oxides, such as iron oxide and aluminum oxide, are not commonly used in sunscreen formulations for several reasons. First, iron oxide, while it can provide some UV protection, is not as effective as titanium dioxide and zinc oxide in blocking both UVA and UVB rays. Additionally, iron oxide can cause skin irritation in some individuals, making it less desirable for sunscreen formulations. Aluminum oxide, on the other hand, is not typically used in sunscreen formulations because it does not provide significant UV protection. It is more commonly used in other applications, such as abrasives and as a component in ceramics, rather than in sunscreens. Overall, titanium dioxide and zinc oxide are preferred in sunscreen formulations due to their effectiveness, safety, and lack of skin irritation, which are qualities that other metal oxides do not always possess.

One of the key advantages of nanoparticles-based sunscreen is that it offers a transparent and lightweight formula. Unlike traditional sunscreens that can leave a white cast on the skin, nanoparticles-based sunscreens are designed to be virtually invisible when applied, providing a more aesthetically pleasing option. Additionally, these sunscreens offer improved photostability, meaning they are less likely to degrade or lose their effectiveness when exposed to sunlight. This ensures that the sunscreen remains active for a longer duration, providing reliable protection throughout sun exposure.^18^ The advantage of being water-resistant, making them suitable for activities like swimming or sweating. They adhere well to the skin and maintain their protective barrier even when exposed to water or perspiration. It is important to note that extensive research has been conducted to ensure the safety of nanoparticles used in sunscreens. Regulatory bodies around the world have approved the use of these nanoparticles in sunscreen products, as they have been found to pose no significant risk to human health when used as directed.^19,20^ This review focuses on the development of chromophore compounds and nanoparticles-based sunscreens and their applications.

## 2. Benzophenone based sunscreen

**Fig. 2:**
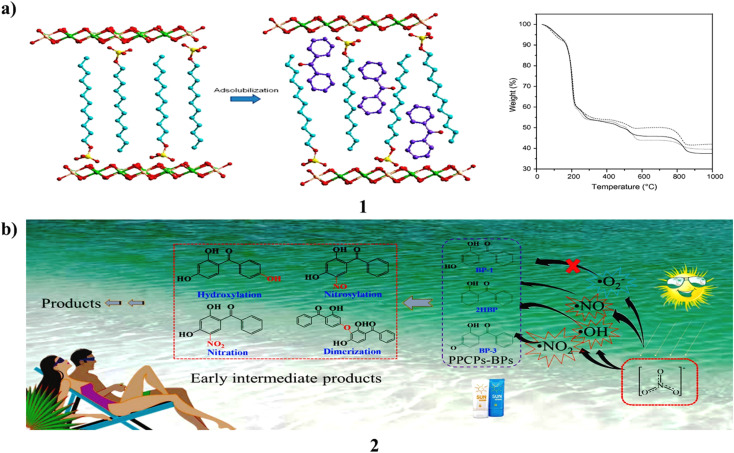
(a) Anionic surfactants of benzophenone-based sunscreens (1) reproduced with permission from ref. 21 Copyright [2013] [Elsevier Publisher]. (b) Photochemical properties of benzophenone sunscreens (2) reproduced with permission from ref. 22 Copyright [2019] [Elsevier Publisher].

A co-precipitation process using alkaline conditions produced layered double hydroxides intercalated with dodecylbenzenesulfonate (1). Following PXRD, FTIR, and TGA/DTA analysis, several processes were used to react the Zn_*x*_Al/SUR compounds with neutral benzophenone. Before and after being exposed to UV light, the products made from benzophenone adsolubilization were examined by PXRD, FTIR, and DRUV-vis spectroscopy. Adsolubilized benzophenone generally had a low content and varied depending on the synthesis process. The microwave irradiation method produced the greatest results, yielding 9.09 weight percent of adsolubilized benzophenone. The products demonstrated strong resilience to UV radiation and good absorption over the whole UV spectrum, from UVC to UVA. They are suitable candidates for the creation of the next generation of sunscreens since they did not result in skin irritation in testing on rabbits (Fig. 2a).^21^ Moreover, Benzophenones (2, BPs) are commonly used ultraviolet filters that have caused a great deal of public worry because of their possible ability to alter the endocrine system. They thoroughly explored the photochemical behavior and destiny of these organisms, which is mediated in aquatic settings by nitrate. The results showed that 2,4-dihydroxybenzophenone had a 31.6% mineralization rate after 12 hours of irradiation and that 10 μM of 3 BPs may be destroyed in 4 hours of simulated solar irradiation in a 10 mM nitrate solution at pH 8.0. Their photolytic rates (*k*_obs_) showed a substantial linear association with the log values of the concentration of nitrate for 0.1–10 mM, and in three real waters, the rates of BP were similarly significantly associated with the intrinsic nitrate content. Additionally, higher transformation rates under alkaline conditions were observed, especially for BP, whose kobs at pH 10 were 8.3 times higher than at pH 6.0. In addition, dissolved oxygen (DO) also has some impact on reaction kinetics. According to quenching experiments, the three reactive oxygen species (ROS), namely ˙OH, ˙NO, and ˙NO_2_, participated in this BP photolysis, and the contribution of ˙OH accounted for 32.1%. The model molecule used to examine the toxicity alterations and transformation routes in this system is BP. Based on examination of liquid chromatography quadrupole time-of-flight mass spectrometry data and density functional theory, four primary transformation pathways hydroxylation, nitrosylation, nitration, and dimerization were postulated. *Photobacterium phosphoreum* found the produced intermediates to be more harmful than the parent BP in the toxicity test. These findings therefore contribute to the elucidation of phototransformation processes and the assessment of the possible ecological hazards associated with BPs in aquatic settings (Fig. 2b).^22^

**Fig. 3:**
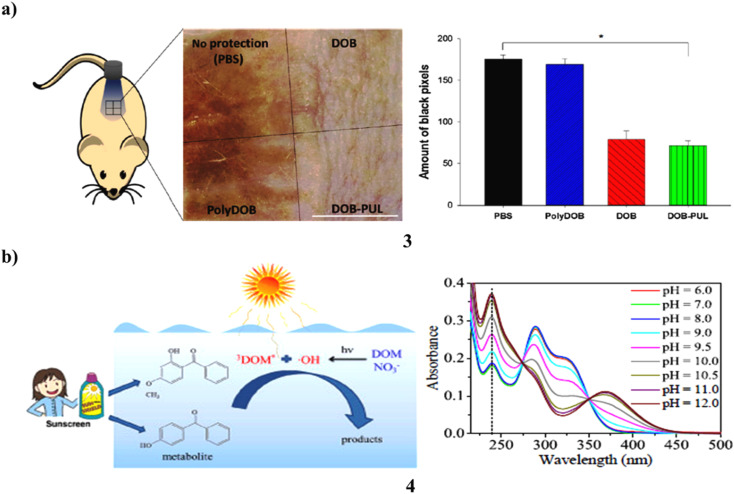
(a) Polysaccharide-benzophenone conjugates of sunscreen protection (3) reproduced with permission from ref. 23 Copyright [2018] [Elsevier Publisher]. (b) Photochemical conversion of sunscreen agent benzophenone-3 (4) reproduced with permission from ref. 24 Copyright [2016] [Elsevier Publisher].

The ingredients in sunscreen have been created to shield skin from UV rays. However, a lot of organic sunscreen ingredients include tiny molecules that are absorbed into the skin of people after topical use, causing systemic negative effects. Use a polymer and an organic sunscreen substance to reduce the side effects of traditional sunscreens. An organic sunscreen chemical called dioxybenzone (3) is chosen, and natural polymer pullulan is used to polymerize it. Dioxybenzone is given a lengthy polymer backbone *via* polymerization, which also maintains the distance between its benzene rings and prevents the photoabsorption intensity from decreasing. UV/vis spectrophotometry proved that the UV absorption patterns of dioxybenzone-pullulan polymer (DOB-PUL) and dioxybenzone (DOB) were identical. The Franz diffusion cell was used to assess the buildup of sunscreen components on the skin and establish that DOB accumulates whereas DOB-PUL does not. Most noteworthy, DOB demonstrated greater plasma concentration than DOB-PUL after numerous administrations (Fig. 3a).^23^ The photodegradation of 4-OH-BP3 and BP-3 (4) was examined in freshwater, ocean, and pure water. The results reveal that neutral forms of BP-3 and 4-OH-BP3 resist photodegradation more than anionic forms do in pure water, and direct photodegradation of both exhibits considerable speciation dependency. In the meantime, both compounds' photoinduced transformation is significantly aided by indirect photodegradation caused by reactive species, particularly DOM. While ^3^DOM* and *OH are primarily responsible for indirect photodegradation in freshwater, it is ^3^DOM* that does so in saltwater (Fig. 3b).^24^

**Fig. 4:**
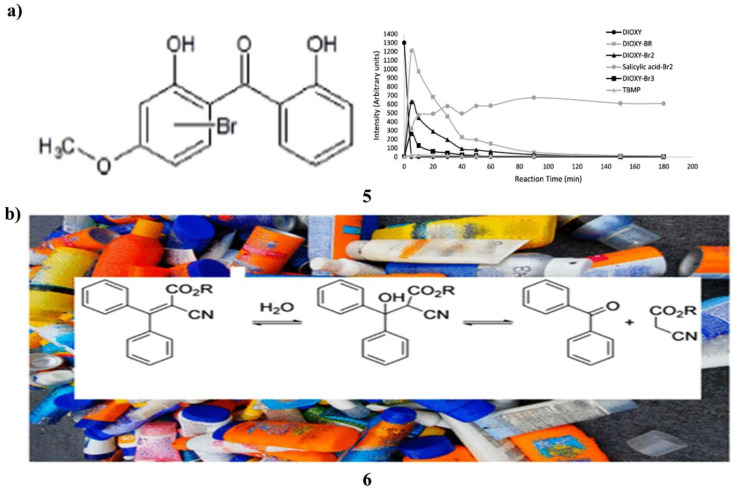
(a) UV filter sunscreen of dioxybenzone (5) reproduced with permission from ref. 25 Copyright [2019] [Elsevier Publisher]. (b) Benzophenone accumulates based sunscreen products (6) reproduced with permission from ref. 26 Copyright [2021] [ACS Publisher].

In chlorinated bromide-rich water, the mutagenicity of four organic UV filters oxybenzone, dioxybenzone, avobenzone, and octyl methoxycinnamate was examined. The Ames test was used to assess the mutagenicity of *Salmonella typhimurium* TA98 without S9 mix. To clarify the mutagenic transformation products, high-resolution mass spectrometry was used in chemical analysis. Only dioxybenzone (5) of the tested UV filters showed a blatant carcinogenic activity after being chlorinated in seawater at a 1 : 10 ratio. When chlorine was introduced at greater concentrations, however, no mutagenic activity was seen. Mutagenic extracts included several brominated dioxybenzone transformation products, according to high-resolution mass spectrometry analyses. The transformation products' time course examination at increasing chlorine dosages revealed that they were unstable and vanished more quickly. Since no transformation products were found this instability explained dioxybenzone did not exhibit mutagenic activity when 1000-fold extra chlorine was applied. Discussion is had over applicable these findings are to the swimming pool environment. To assess the total effect of high amounts of chlorine on the overall mutagenicity, more research is required that takes into account the mutagenicity of both the final disinfection byproducts and the intermediate transformation products. This study emphasizes crucial it is to take into account organic UV filters' reactivity and the compounds they turn into while creating sunscreen formulas in cleaned recreational waters (Fig. 4a).^25^ Additionally, benzophenone (6) is an endocrine disruptor, mutagen, and carcinogen. In the US, it is forbidden to have it in food or food packaging. Benzophenone is not allowed in any skincare products, including sunscreen, anti-aging creams, and moisturizers, under California Proposition 65. This study set out to find out whether benzophenone was a common ingredient in a variety of commercially available sunscreens with sun protection factors/sunscreen products, whether its concentration increased over time, and whether octocrylene degradation was most likely the source of benzophenone contamination. Eight commercial sunscreen products from the United States and nine from the European Union were each tested for benzophenone content in triplicate. Two sources of octocrylene were tested with only one component. The Food and Drug Administration of the United States accelerated stability aging technique was used to test these identical SPF products for 6 weeks. In the items that had aged more quickly, benzophenone was detected. Recent acquisitions of sixteen octocrylene-containing product lines exhibited an average benzophenone content of 39 mg kg^−1^, ranging from 6 mg kg^−1^ to 186 mg kg^−1^. It was not detectable in the product that did not contain octocrylene. After subjecting the 17 products to the U.S. FDA-accelerated stability method, the 16 octocrylene-containing products had an average concentration of 75 mg kg^−1^, ranging from 9.8 mg kg^−1^ to 435 mg kg^−1^. The substance that did not include octocrylene did not contain any benzophenone at all. The produced component for pure octocrylene contained benzophenone. Octocrylene undergoes a retro-aldol condensation to produce benzophenone. In real life, the skin may absorb up to 70% of the benzophenone included in these sunscreen creams. The U.S. FDA has created a zero-tolerance policy for the food ingredient benzophenone. In 2019, there were 2999 SPF products with octocrylene sold in the US. The efficacy of octocrylene as a benzophenone generator in SPF or other consumer products should be swiftly assessed by regulatory bodies (Fig. 4b).^26^

**Fig. 5:**
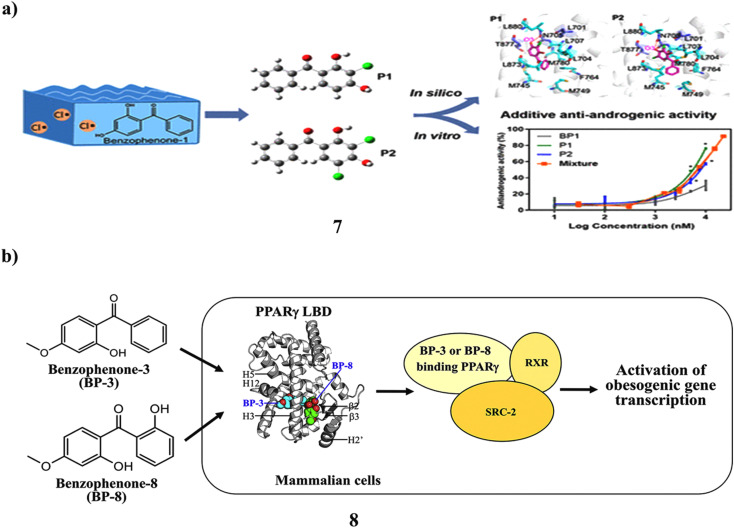
(a) Molecular dynamics simulations study of benzophenone-1 (7) reproduced with permission from ref. 27 Copyright [2021] [ACS Publisher]. (b) Obesogenic activity of benzophenone-3 and benzophenone-8 (8) reproduced with permission from ref. 28 Copyright [2020] [Elsevier Publisher].

The frequent detection of traces of benzophenone-1 (7) in recreational and environmental waterways has raised public concern. Its sensitivity to lingering chlorine and its ability to cause endocrine disruption as a result is unclear. They looked into the chlorination content of BP-1 in water from swimming pools and assessed the impact on the human androgen receptor's (AR) endocrine system. Mass spectrometry and NMR correlation spectroscopy were used to distinguish between and describe the mono- and dichlorinated product structures. In yeast two-hybrid experiments, it demonstrated noticeably more antiandrogenic efficacy compared to BP-1 (12.89 μM). Although increased hydrophobic interactions are primarily responsible for improved affinity for binding between chlorine-based products and the AR ligand binding domain, the second form of chloride in P2 still impairs the complex motion due to the solvation penalty, according to additional energy calculations. The protein dynamics were shown to be in a long-timescale equilibrium by the 350 ns Gaussian accelerated molecular dynamics simulations. According to the concentration addition model, the combination of BP-1, P1, and P2 triggered additive antiandrogenic action. NKX3.1 and KLK3 are AR-regulated genes, and P1 and P2 at 1 μM reduced their mRNA expression by 1.7–9.1-fold in androgenactivated LNCaP cells. Because residual chlorine in aquatic settings naturally chlorinates BP-1 findings on increased antiandrogenic activity and disrupted AR signaling offered proof connecting the use of personal care items with possible health problems (Fig. 5a).^27^ However, the UV filter components in many sunscreen lotions include benzophenone-8 (BP-8) and benzophenone-3 (8, BP-3). In the adipogenesis model using the bone marrow of human mesenchymal stem cells (hBM-MSCs), the long-wave UV A filter avobenzone's obesogenic action was clarified. Due to the chemical similarities between BP-3 and BP-8 and avobenzone, the obesogenic potentials of these compounds were examined in this work. More effectively than avobenzone, BP-3 and BP-8 stimulated the release of adiponectin during adipogenesis in hBM-MSCs. Both BP-3 and BP-8 are directly attached to the peroxisome proliferator-activated receptorγ (PPARγ) during target identification, which was accompanied by the recruitment of the steroid receptor coactivator-2 (SRC-2). While BP-8 was a partial PPARγ agonist, BP-3 worked as a complete PPARγ agonist. In addition, human epidermal keratinocytes, a key target of UV filters in human skin, greatly boosted the gene transcription of PPARα, PPARγ, and important lipid metabolism-associated enzymes. They are obesogenic environmental substances like organotins, phthalates, and bisphenols (Fig. 5b).^28^

**Fig. 6:**
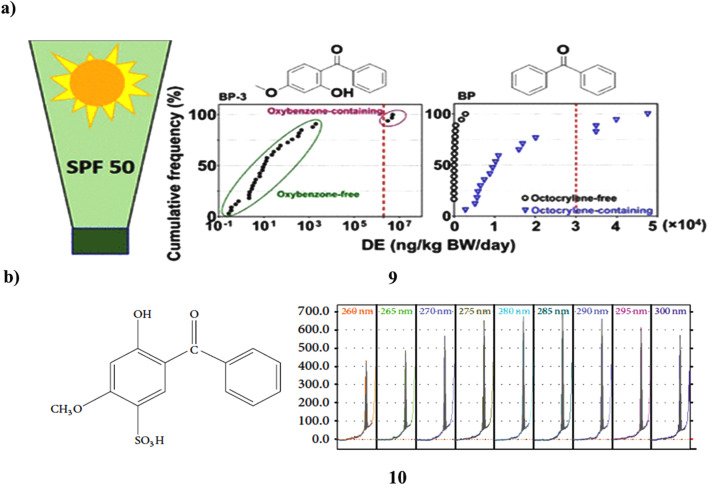
(a) Benzophenone derivatives of UV filters in sunscreen products (9) reproduced with permission from ref. 29 Copyright [2022] [ACS Publisher]. (b) Hydrophilic interactions of benzophenone-4 based sunscreen (10) reproduced with permission from ref. 30 Copyright [2015] [Hindawi Publisher].

The UV filters of the benzophenone (9) class are estrogenic substances that are widely utilized in sunscreen products, raising concerns about human exposure. In 50 items from 44 brands that were offered in the United States in 2021, 14 BP UV filters were tested to measure exposure to BP derivatives in sunscreens. It was found in around ≥70% of the samples. The 50 items had a geometric mean (GM) concentration of 6600 ng g^−1^ for the total of these BPs (∑_14_BPs). Its content in oxybenzone-containing goods was 5–6 orders of magnitude greater than in “oxybenzone-free” items, making it the predominant BP in those products. Even those goods with the label “oxybenzone-free” had it in greater than 90% of the samples tested. Octocrylene-containing goods had concentrations that were around 100 times greater than “octocrylene-free” products (GM: 15 900 *vs.* 151 ng g^−1^). Dermal exposure dosages of BP-3 from goods containing oxybenzone (GM: 4 140 000 ng per kg body weight (BW) per day) and BP from certain (24%) items containing octocrylene (GM: 12 200 ng per kg BW per day) were above reference levels (2 000 000 and 30 000 ng per kg BW per day for BP-3 and BP, respectively). This study shows that BP and BP-3 concentrations in sunscreen creams vary considerably and may be significant even in items marked as being free of oxybenzone or octocrylene, raising ongoing concerns about dermal exposure (Fig. 6a).^29^ Thin-layer chromatography was used to extract benzophenone-4 (10, BZ4) from hair shampoo's surfactants, colors, preservatives, and other ingredients. The stationary phase was silica gel 60, while the mobile phase was an ethyl acetate–ethanol–water-pH 6 phosphate buffer. At 285 nm, chromatograms were scanned using densitometry. BZ4's densitometric calibration curve has a nonlinear shape and an *R* > 0.999 value. Approximately 0.03 and *ca.* 0.1 μg per spot, respectively, served as the detection and quantification limits. The outcomes of UV spectrophotometry using the zero and second derivatives were contrasted with those of HPTLC-densitometry. Calibration curves for spectrophotometric techniques were linear with *R* > 0.9998. The chromatographic technique received full validation (Fig. 6b).^30^

**Fig. 7:**
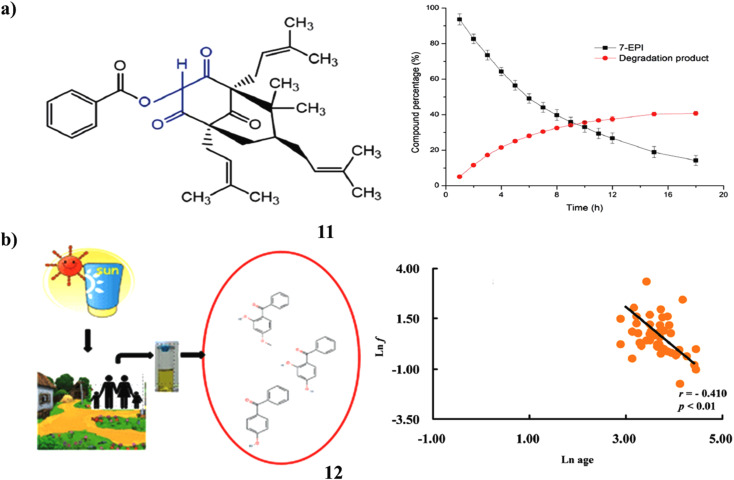
(a) LC-UV study of prenylated benzophenone (11) reproduced with permission from ref. 31 Copyright [2019] [RSC Publisher]. (b) Urinary benzophenone-type of UV filters in sunscreen (12) reproduced with permission from ref. 32 Copyright [2018] [RSC Publisher].

Potential therapeutic candidate 7-*epi*-clusianone (11, 7-EPI) is a naturally occurring prenylated benzophenone that is isolated from the fruits of *Garcinia brasiliensis*. A designed and approved stability-indicating technique by LC-UV was used to assess the benzophenone's intrinsic stability. One significant oxidative degradation product was found, and 7-EPI degradation under forced oxidation followed first-order kinetics. Following a reaction in a Baeyer–Villiger-type method, this novel compound's structural elucidation revealed that one atom of oxygen was stabilized by a resonant between two carbonyl moieties. The prenylated benzophenone, found as 7-*epi*-oxi-clusianone, may be investigated as a possible therapeutic candidate or sunscreen ingredient (Fig. 7a).^31^ A class of compounds known as benzophenone (12)-type UV filters are frequently employed in sunscreen products to stop UV radiation from damaging human skin. They have also been investigated as endocrine disruptors, hepatotoxic, and pneumotoxic toxicants *in vitro* and *in vivo*. Large cities in China were the focus of research on human exposure to BPs, whereas rural regions were disregarded. In Guangdong Province, China, this study evaluated and compared the urine concentrations of five BPs. Additionally investigated were the correlation patterns and composition profiles of various BPs. They recommended high levels of BP-3 and 4-OH-BP exposure in rural regions. The concentrations of urine BP-1 and 4-OH-BP showed significant positive associations, as did those between urinary BP-1 and BP-3. This study addressed several crucial issues for estimating human exposure and gave crucial data for calculating the health hazards and BP exposure for rural residents (Fig. 7b).^32^

**Fig. 8:**
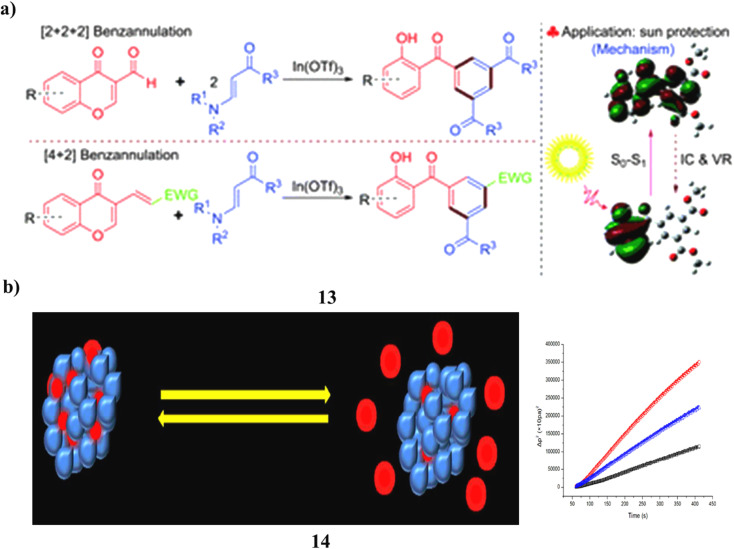
(a) Sun protection of indium(iii)-catalyzed benzannulation (13) reproduced with permission from ref. 33 Copyright [2016] [RSC Publisher]. (b) Skin protection of benzophenone-3 based ZnO nanoparticles (14) reproduced with permission from ref. 34 Copyright [2013] [RSC Publisher].

The highly regioselective [2 + 2 + 2] benzannulation of 3-formylchromones with β-enamino esters, indium(iii)-catalyzed synthesis of various and functionalized 2-hydroxybenzophenone derivatives (13), excellent to good yields were produced. A domino Michael/retro-Michael/6π-electrocyclization/deformylation reaction drives the progression of this benzannulation process. Additionally, 3-substituted chromen-4-ones and β-enamino esters were combined to form 2-hydroxybenzophenones by a [4 + 2] benzannulation process that was catalyzed by indium(iii). In addition, the properties of the UV-vis spectrum of produced 2-hydroxybenzophenones were studied about substituents and π conjugation. Compared to the most used sunscreen ingredient, oxybenzone, it demonstrated greater UV protection activity (Fig. 8a).^33^ ZnO NPs, on the other hand, were created specifically to entrap Bp-3 (14) and demonstrated recurrent on-demand release, encapsulation, and UV radiation sensitivity as well as minimal cytotoxicity to skin cells. Potential sunscreen uses for the Bp-3-loaded ZnO NPs exist (Fig. 8b).^34^

**Fig. 9:**
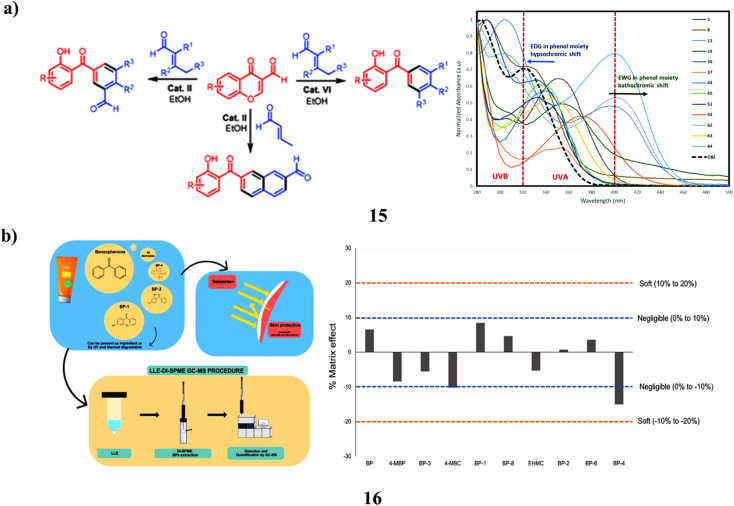
(a) UV-A/B filters study of cascade benzannulation (15) reproduced with permission from ref. 35 Copyright [2020] [RSC Publisher]. (b) Direct-immersion solid-phase microextraction of benzophenone derivatives (16) reproduced with permission from ref. 36 Copyright [2023] [Elsevier Publisher].

The 3-formylchromones and, α,β-unsaturated aldehydes as the starting materials, they created an environmentally friendly organocatalyst-controlled technique for the highly selective synthesis of polyfunctionalized 2-hydroxybenzophenone frameworks (15), which include 2-hydroxy-3′-formylbenzophenones. The unique procedure makes use of organocatalysts that are safe for the environment, easily accessible, affordable, non-toxic, and operationally straightforward. The newly created compounds were effectively used in C–H alkenylation and alkylation processes to create novel and intriguing materials for biology. Comparing the created molecules to the widely accessible sunscreen component oxybenzone, they demonstrated higher photoprotective characteristics (Fig. 9a).^35^ Since benzophenones (16) are efficient UVA and UVB filters, they are often utilized in industry. In Europe, sunscreen products are required to contain benzophenone-3, commonly in combination with additional filters like octocrylene. They must be monitored since UV light can make them mutagenic, and octocrylene may turn into BPs. To separate and identify BPs in sunscreen products with possible outcomes, liquid–liquid extraction was then followed by direct-immersion microextraction in the solid phase (LLE-DI-SPME). The most efficient SPME fiber was found to be polyacrylate fiber after factors such as extraction solvent, pH, adsorption, desorption duration, stirring, sating effect, and the presence of organic solvents were adjusted. Gas chromatography-mass spectrometry was used for detection and quantification. The linear range ranged from 0.16 to 2000 μg kg^−1^, whereas the analytical parameters' limits of detection were 0.05 to 0.10 μg kg^−1^. The method's recovery varied from 83 to 103%, and its precision of 3.2 to 18.7% relative standard deviation (RSD) was good without showing much of a matrix impact. The DI-SPME approach was challenging and the samples were complicated, but the method held up well. The suggested approach effectively identified 10 BPs in 6 separate sunscreen creams. Sunscreens included a total of 165 to 931 mg kg^−1^ of BPs, with BP-3 being found in all samples at levels ranging from 4.2 to 740 mg kg^−1^ (Fig. 9b).^36^

**Fig. 10:**
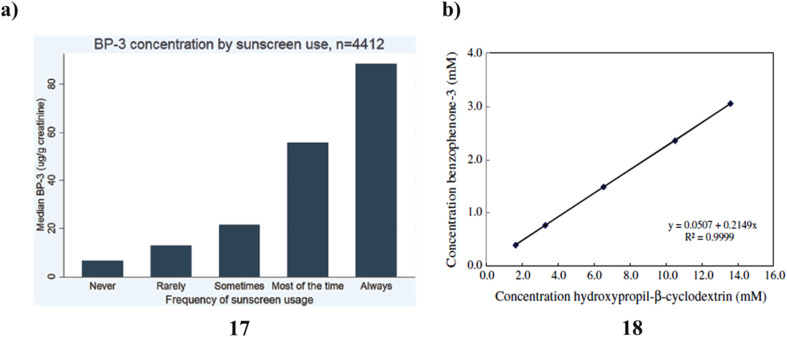
(a) Sunscreen application of urinary benzophenone-3 (17) reproduced with permission from ref. 37 Copyright [2015] [Elsevier Publisher]. (b) Photoacoustic spectroscopy study of benzophenone-3-hydroxypropyl-β-cyclodextrin (18) reproduced with permission from ref. 38 Copyright [2011] [Elsevier Publisher].

Across all age categories, sexes, and racial/ethnic groupings, there is a positive correlation between self-reported frequency of sunscreen usage and urine BP-3. Although these findings indicate a significant relationship between self-reported use and the BP-3 (17) biomarker for actual sunscreen utilization, more research will be required to determine whether it is possible to enhance the biomarker for actual use as well as validate self-reported sunscreen use through more specific questions about the amount of sunscreen used, the number of days used each week, frequently sunscreen is applied again during the day, and the typical SPF used (Fig. 10a).^37^ Thermal analysis and PAS revealed that BZ-3 (18) and HPCD would be complex in a 2 : 1 stoichiometric ratio. Histological examination revealed no tissue reactivity when formulations containing the complex were used. Sunscreen penetration was similarly minimal according to PAS, which is consistent with the outcomes of using PAS. As a result, it is advantageous to employ the BZ-3-HPCD complex in sunscreen compositions. Additionally, PAS is a technology that may be used with other, more traditional methods to examine CDs create inclusion complexes, and deeply the complexes penetrate the skin. Given these findings, it can be concluded that the formulation containing the complex BZ-3-HPCD is a good option for enhancing the effectiveness of sunscreen compositions and that PAS may be a valuable method for assessing the UV sensitivity of these formulations (Fig. 10b).^38^

Benzophenone-based sunscreens offer broad-spectrum protection against UVA and UVB rays, making them effective at preventing sunburn and skin damage. However, some benzophenones have been associated with potential hormone-disrupting effects and environmental concerns, leading to their restricted use in certain regions and formulations.

## 3. Flavanoid based sunscreen

**Fig. 11:**
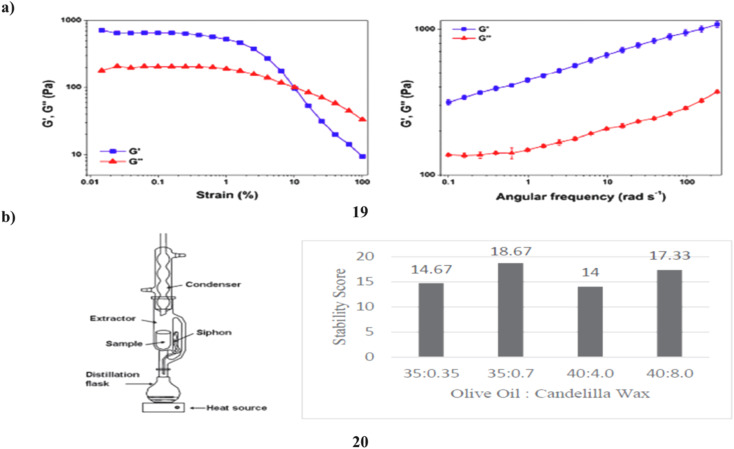
(a) Sunscreen formulation of flavonoid compounds (19) reproduced with permission from ref. 39 Copyright [2019] [MDPI Publisher]. (b) SPF values of *Spirulina platensis* for flavonoids (20) reproduced with permission from ref. 40 Copyright [2020] [AIP Publisher].

The phytocosmetic (19) sunscreen emulsion has antioxidant properties and a combination of plant extracts high in flavonoids. Sun protection elements, antioxidant activity, skin sensitivity, photostability, cutaneous permeability, and flavonoid retention were assessed *in vitro*. Following the loading of the extract mixture, thermodynamically stable emulsions were produced and examined for sensory analysis. When kept at low temperatures, the emulsion was stable; nevertheless, after 120 days, the concentrations of quercetin and rutin, which were 2.8 ± 0.39 μg mL^−1^ and 30.39 ± 0.39 μg mL^−1^, respectively, were over their limits of quantification. A standard topical product was found to have equal spreadability, low rupture strength, and adhesiveness. The discovered phytocosmetic sunscreen also showed higher pseudo-plastic, viscoelastic, and brittleness characteristics. The product demonstrated an essential wavelength of 387.0 nm and an UV rays both A and B (UVA/UVB) efficiency of 0.78, demonstrating that the produced formulation has UVA/UVB protection capability, defending skin against UV radiation-related damage. Rutin was demonstrated to pass through the skin's physical barrier and measured in the stratum corneum (3.27 ± 1.92 μg mL^−1^) by a tape stripping and retention test (114.68 ± 8.70 μg mL^−1^). By using an *in vitro* assay, the developed flavonoid-enriched phytocosmetic was shown to be non-irritating to the skin (Fig. 11a).^39^ The number of natural ingredients used as active agents for sunscreen is constantly growing, and one of them is microalgae, specifically *Spirulina plantesis* (20), a cyanobacterium that has naturally absorbed UV compounds, particularly flavonoids, in its cells. Due to its capacity to raise the SPF and absorb the highest wavelength of UV rays, flavonoid has the potential to be employed as an active component in sunscreen. To get the best cream stability and SPF ratings from sunscreen cream formulations, it is varied in the range of 1–10% w/w and the ratio of olive oil to candelilla wax was also adjusted, with values of 10 : 1 and 5 : 1. Based on the results, the total flavonoid compound in the dry and fresh microalgae foundation samples was determined to be 22.10 mg g^−1^ and 10.91 mg g^−1^, respectively. The best sunscreen formulation in this study had 7% (w/w) microalgae extract and a 35 : 7 ratio of olive oil to candelilla wax. This formulation has a strong stability score (17.33 out of 20) and a good SPF rating (29.06), which is classified as ultra-SPF. Because the total microorganisms were still below the necessary total microbial of SNI and did not irritate the skin, the flavonoid-containing sunscreen derived from microalgae extract is safe to use (Fig. 11b).^40^

**Fig. 12:**
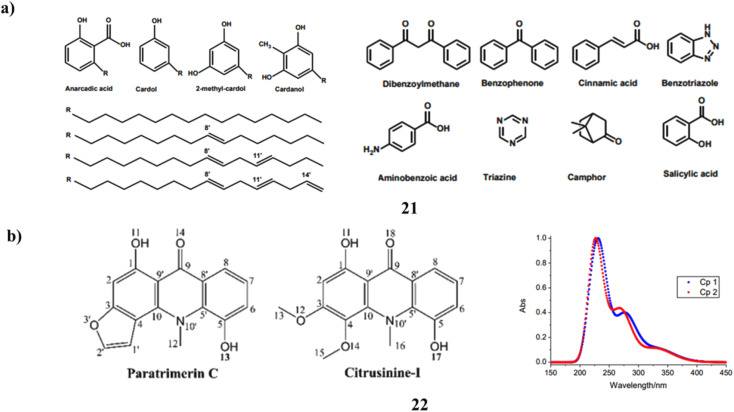
(a) Cashew Nutshell liquid of sun protection (21) reproduced with permission from ref. 41 Copyright [2022] [MDPI Publisher]. (b) Photoprotective properties of acridone derivatives (22) reproduced with permission from ref. 42 Copyright [2020] [Wiley Publisher].

Natural phenolic chemicals may be found at low cost in cashew nutshell liquid (21, CNSL), which has a wide range of uses. These synthetic UV filters, which are prevalent in commercialized sunscreen products, contain chromophores with chemical structures identical to those of these phenolic compounds. In this study, the effects of solvents on crude CNSL's yield, total phenol content (TPC), total flavonoid content (TFC), and sun protection factor were examined. Hexane had the lowest yield (30.4 ± 0.7%), whereas ethanol had the greatest (49.3 ± 3.2%), according to the percent yield range. The findings showed that solvent extraction significantly affects the yield and SPF of CNSL. Since it has an excellent TPC and SPF, it could be the optimum solvent for extracting CNSL (Fig. 12a).^41^ DFT and TD-DFT at the M05-2X/6-311++G(3df, 3p)//M05-2X/6-31+G(d) level of theory have been used to investigate the photoprotective characteristics of two naturally occurring acridone derivatives (22). In the gas phase, water, and pentyle thanoate, three typical pathways defined for the antioxidant characteristics, including H-atom transfer (HAT), proton transfer (PT) towards HOO*/HO* radicals, and single electron transfer (SET) were examined. According to the DFT results, both compounds effectively scavenge HOO* and HO* radicals in all mediums using the HAT mechanism. The most preferred reaction for the HO* radical in water is the HAT reaction (Δ*H* −37.7 kcal mol^−1^). Additionally, TD-DFT was used to clarify the examined compound's effective UV-absorption capability. All substances can absorb UV rays in the 200–335 nm range, with the simplest excitations occurring between 334 and 332 nm and the highest absorptions occurring between 234 and 227 nm. The equivalent UV-absorption is given the HOMO to LUMO and HOMO-3 to LUMO (π–π*) transitions (Fig. 12b).^42^

**Fig. 13:**
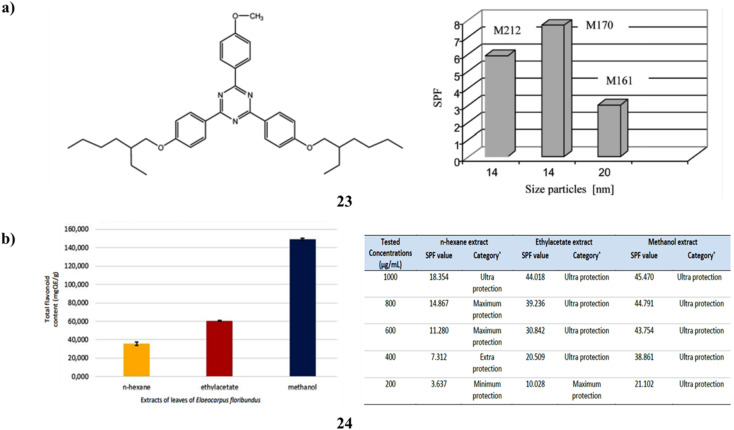
(a) Photoprotection of dermal tissues for BEMT (23) reproduced with permission from ref. 43 Copyright [2008] [Taylor & Francis Publisher]. (b) Sunscreen activity of *E. floribundus* blume (24) reproduced with permission from ref. 44 Copyright [2023] [FIP Publisher].

Two to eight percent of the naturally occurring antioxidant flavonoids (23) are added. Sun protection factor measurement and connection with antioxidant activity have been used to monitor the photoprotective properties of creams (three kinds of TiO_2_ NPs (UV-Titan M161 and M212 and M170) produced by KEMIRA) *in vitro*. During the irradiation process, the mixtures of TiO_2_ + flavonoids produce the amplification of the SPF value because of the photocatalytic impact of the TiO_2_ pigment and bis-ethylhexyloxiphenol-metoxiphenyltriazine (BEMT), which is tested into the collagen base. Both UVA and UVB photoprotection are provided by the cream's combination of TiO_2_, BEMT, and flavonoids (Fig. 13a).^43^ Additionally, *Elaeocarpus floribundus* (24) blume leaves have long been utilized as a remedy for several illnesses; its hot water infusion is used as a gargle to heal sore gums and alleviate rheumatoid arthritis. Furthermore, it has demonstrated that the total phenolic content of its methanol extract is very high. Comparing the several extracts, the *E. floribundus* blume leaf methanol extract showed the highest amount of TFC and the greatest potential for sunscreen action (Fig. 13b).^44^

**Fig. 14:**
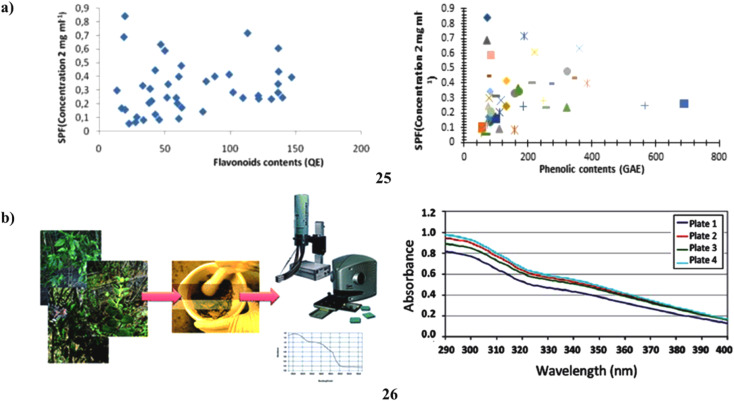
(a) Sun protection factor of flavonoid compounds (25) reproduced with permission from ref. 45 Copyright [2019] [Pharmacotherapy Group Publisher]. (b) Sunscreen formulation of Brazilian *Lippia sericea* (26) reproduced with permission from ref. 46 Copyright [2014] [RSC Publisher].

To assess the relationship between the amount of phenol and flavonoid content and antioxidant activity and the sun protection factor. According to this study, there is a link between SPF and phenolic and flavonoid concentration. Sunscreen compositions can employ the adequate SPF provided by an ultrasonically aided extract of *C. melo* leaf (25) (Fig. 14a).^45^ Moreover, a natural UV filter may be employed in the formulation as a single broad-spectrum sunscreen from the *Lippia* species (26). To calculate their UVB protection factor, the UV transmission of sunscreens generated from four distinct *Lippia* species was first measured. Next, using diffuse transmittance spectroscopy, the *in vivo* SPF as well as the *in vitro* UVA radiation shielding factor (UVAPF) of the extract from the species with the best results were determined. The *in vitro* SPF ratings for the natural sunscreens ranged from 1.7 to 7.6. The *L. sericea* species provided the highest SPF; when employed as a single UV filter in a lotion, it had an *in vivo* SPF of 7.5 and a UVAPF of 2.97. It was discovered that the plant's overall polyphenolic content, rather than its flavonoid or antioxidant capacity, is what gives this sunscreen its photoprotective properties. As a consequence, the findings of this study showed that *L. sericea*'s natural sunscreen may someday find commercial utility (Fig. 14b).^46^

**Fig. 15:**
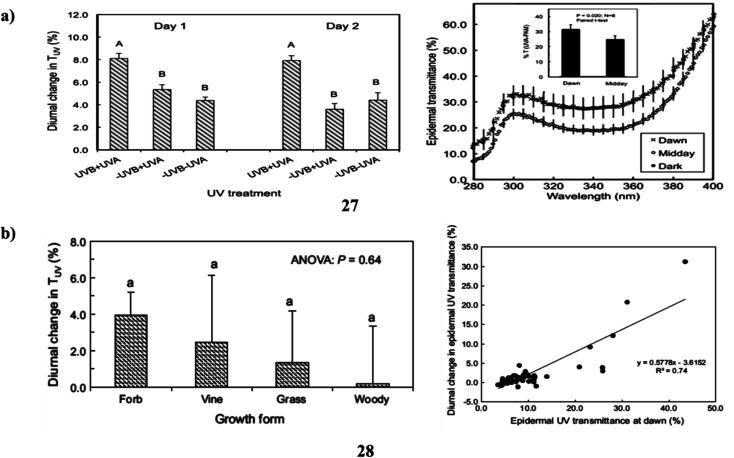
(a) Solar ultraviolet radiation of flavonoids (27) reproduced with permission from ref. 47 Copyright [2016] [Wiley Publisher]. (b) Ultraviolet sunscreen protection of flavonoids (28) reproduced with permission from ref. 48 Copyright [2016] [Springer Publisher].

In addition, flavonoids and related phenylpropanoids, which accumulate as ultraviolet-absorbing compounds (27) and cause a decrease in the epidermal UV transmittance (TUV), are the main defenses used by plants against potentially harmful solar UV radiation. These compounds are also essential parts of the overall acclimation reaction of plants to shifting solar UV environments. It is entirely unknown if plants can modify their UV sunscreen defense in reaction to abrupt variations in UV, which happen on a daily basis. The demonstrate that plants may modify their UV-screening characteristics between minutes to hours and that UV radiation is a portion of what causes these changes. Large (30–50%) and reversible alterations in TUV occurred during the day for the domesticated species *Abelmoschus esculentus*, and these modifications were linked to variations in the amounts of various quercetin glycosides and whole-leaf UV-absorbing compounds. Similar findings were found for two more species (*Vicia faba* and *Solanum lycopersicum*), however, *Zea mays* showed no such alterations. These findings have practical implications for using UV to increase crop vigor as well as quality in controlled environments. They also raise important questions about the expenses and advantages of UV-protection strategies in plants (Fig. 15a).^47^ The ultraviolet-absorbing substances (28) (flavonoids and related phenylpropanoids) in higher plants' epidermis reduce the diffusion of solar UV radiation to underlying tissues and serve as a key mechanism of acclimation to shifting UV conditions brought on by ozone depletion and climate change. A gradient of ambient solar ultraviolet and climate is represented by the screening species of diverse wild and cultivated plants growing in four different places. Non-destructive studies of adaxial TUV revealed that there was significant interspecific heterogeneity in the amplitude of these changes and that midday declines in TUV occurred in 49% of the species studied, encompassing both herbaceous and woody growth types. Overall, *Louisiana* plants showed more diurnal fluctuations in TUV than plants in the other locales. The extent of these alterations was also strongly linked with the lowest daily air temperatures across all taxa, but not with daily UV irradiances. The findings show that diurnal variations in UV shielding are common in higher plants, vary across and within species, and are often largest in herbaceous plants that thrive in warm climates. These findings imply that plant species have different “strategies” for protecting themselves from UV radiation, albeit it is still unknown the functional and ecological implications of these differences in UV sunscreen protection (Fig. 15b).^48^

**Fig. 16:**
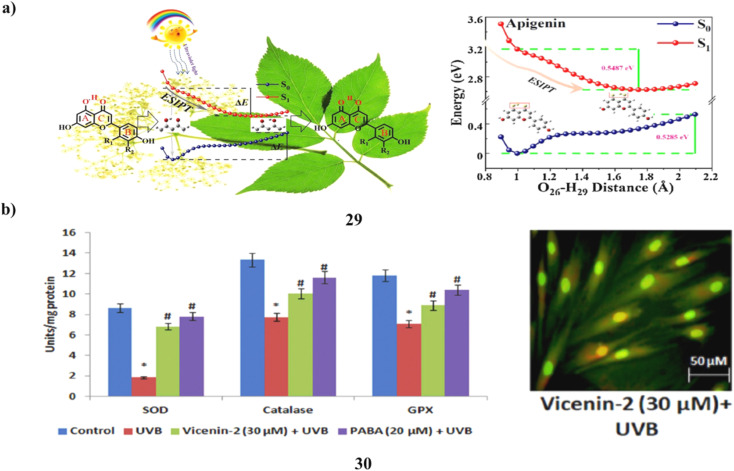
(a) ESIPT mechanism of flavonoids (29) reproduced with permission from ref. 49 Copyright [2021] [Elsevier Publisher]. (b) Sunscreen properties of vicenin-2 ameliorates (30) reproduced with permission from ref. 50 Copyright [2019] [Elsevier Publisher].

The creation and investigation of three flavonoid (29) sunscreen formulations was successful. It was discovered sunscreen chemicals undergo excited state intramolecular proton transfer using steady-state spectroscopy and time-dependent density functional theory. The estimated UV-vis absorption spectra and fluorescence emission spectra accord well with the outcomes of the methanol solution experiments. The potential energy curve shows that the three sunscreen chemicals lack of an energy barrier makes the ESIPT procedure simple to carry out. Therefore, the excitation energy that was absorbed might return to the ground state *via* a non-radiative relaxing process. The three flavonoids can function as sunscreens, according to light stability testing. In addition to serving as a theoretical foundation for the creation of new sunscreen compounds, it is a process in sunscreen processes (Fig. 16a).^49^ The main environmental element that contributes to erythema, inflammation, photoaging, and skin carcinogenesis is exposure to UV radiation. Vicenin-2 (30) is a bioflavonoid that has been identified from a number of therapeutic plants. The impact of vicenin-2 on UVB-linked oxidative stress and photoaging signaling in human dermal fibroblasts (HDF). HDF cells went into apoptosis as a result of UVB-irradiation's markedly increased levels of intracellular ROS, lipid peroxidation, DNA damage, and antioxidant depletion. Intriguingly, vicenin-2 was administered to HDF cells 1 hour before UVB exposure to suppress the production of ROS, TBARS, apoptosis, and DNA damage. Oxidative stress and photoaging are associated with MAPKs and MMP signaling, which are thought of as photoaging and differentiation. It stops the overexpression of MAPKs and MMPs in HDF cells upon UVB exposure. Due to its sunscreen-like qualities, it could be a potential bioactive element to absorb UV photons and shield the skin cells from UVB-related oxidative stress and photoaging signals (Fig. 16b).^50^

Flavonoid-based sunscreens offer the advantage of being natural compounds with antioxidant properties, potentially providing additional skin benefits beyond UV protection. However, their effectiveness as broad-spectrum UV blockers and their stability in sunscreen formulations may be limited compared to synthetic UV filters, which could impact their overall sun protection capabilities.

## 4. Polymeric nanoparticles-based sunscreen

**Fig. 17:**
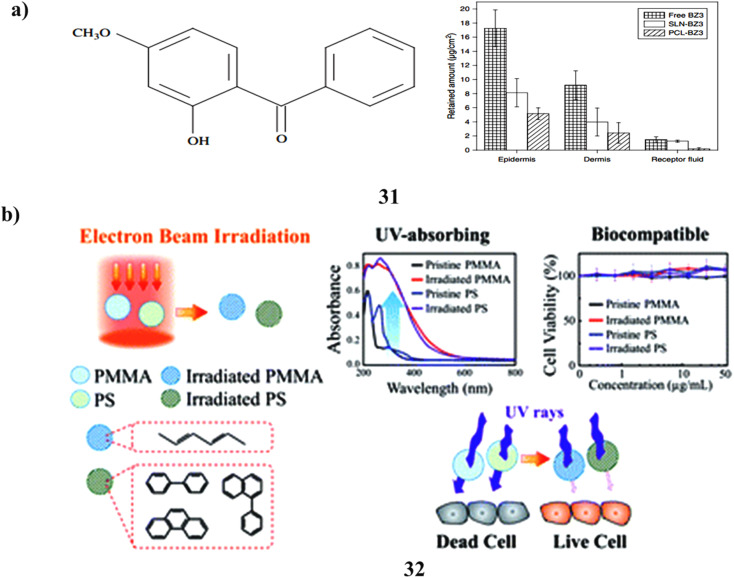
(a) Skin permeation of nanostructured polymer (31) reproduced with permission from ref. 51 Copyright [2011] [American scientific Publisher]. (b) Sunscreen application of polymer nanoparticles (32) reproduced with permission from ref. 52 Copyright [2020] [RSC Publisher].

Benzophenone-3 is to be carried by polymeric nanoparticles (31) that have been prepared and analyzed. By raising the sun protection factor lowering BZ3 penetration into the skin, and lowering BZ3 levels in sunscreen formulation, sunscreen products can be made safer. By using the heated high-pressure homogenization process, BZ3 has been embedded in solid lipid nanoparticles (SLN) and poly(epsilon-caprolactone) (PCL) nanoparticles *via* the nanoprecipitation method. For forty days, the particles remained steady. Compared to BZ3 enclosed in SLN, BZ3 encapsulated with PCL nanoparticles was released more quickly. The encapsulation of BZ3 in both nanostructures improved the sun protection factor. However compared to SLN-BZ3, PCL nanoparticles containing BZ3 decreased its skin penetration more. Additionally, BZ3 in SLN did not exhibit any cytotoxic or phototoxic effects on BABL/c 3T3 fibroblasts or human keratinocytes (HaCaT cells). However, PCL nanoparticles containing BZ3 indicated the potential for phototoxicity in HaCaT cells. Despite this, mice did not develop allergic reactions to BZ3, whether it was present in free form or enclosed in PCL nanoparticles or SLN. The findings imply that these nanostructures could make intriguing sunscreen carriers (Fig. 17a).^51^ A new method based on electron irradiation of polymethyl methacrylate and polystyrene (PMMA-PS NPs) (32) is given for creating non-toxic active ingredients for sunscreens.^52^ Under electron radiation, aromatic rings in PS and conjugated aliphatic C=C rings in PMMA are formed, imparting UV-absorbing properties to the polymers. The extent of conjugation increases with higher electron fluence, leading to a redshift in the absorption spectra. The bombarded polymer NPs' *in vitro* SPF and PA values demonstrate their strong photostability and remarkable UV-absorbing capabilities throughout a wide UV spectrum. Based on OECD TG 432, the irradiated polymer NPs show no discernible evidence of cytotoxicity or phototoxicity and are categorized as nonphototoxic compounds. The electron irradiation process enables the large-scale production of non-toxic, UV-absorbing nanoparticles. Consequently, this method provides a valuable means of developing safe sunscreen ingredients as alternatives to current compounds that pose safety issues. Furthermore, the technique can be employed to manufacture photoprotective personal care items, UV-resistant textiles, coatings with UV protection, and filters for blue light (Fig. 17b).

**Fig. 18:**
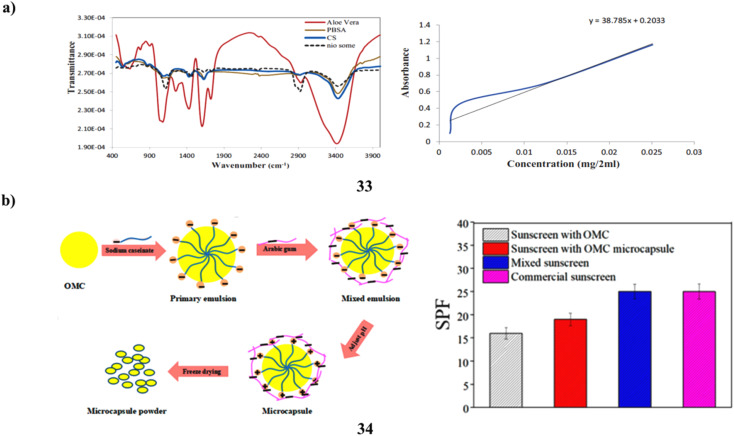
(a) Sunscreen protection of polymer nanoparticles (33) reproduced with permission from ref. 53 Copyright [2022] [SAGE Publisher]. (b) Sunscreen enhancement of biopolymers (34) reproduced with permission from ref. 54 Copyright [2021] [MDPI Publisher].

Furthermore, one of the most hazardous things that can damage skin is UV radiation. The development of sunscreens that effectively shield skin from overexposure to UV radiation is constantly progressing. Phenylbenzimidazole-5-sulfonic acid (PBSA) is typically employed as a sunblocking agent; nevertheless, it has the drawback of photodegrading and potentially damaging cells. To create a carrier polymer with unique and powerful capabilities, PBSA was first encapsulated into niosomes nanoparticles (33) and subsequently coated with chitosan-*aloe vera* (CS-nio-aloe/PBSA). The breakdown of PBSA and epidermal penetration are regulated by this polymer. Fourier transform infrared spectroscopy, scanning electron microscopy, transmission electron microscopy, and dynamic light scattering were used to characterize the CS-nio-aloe/PBSA polymer nanoparticles. Mice skin was used to measure the epidermal transparency of coated PBSA and investigate the carrier polymer release rate *in vitro.* The sunscreen-containing nanoparticle polymer was successfully produced, exhibiting an 80% encapsulation efficiency. The skin's surface was entirely covered in the formulation (CS-nio-aloe/PBSA). This bolsters its application as a skin protector, and its nanostructures prolong the release of PBSA. Improved cellular preservation, UV protection, management of free PBSA, and restricted penetration into the mouse skin epidermis may all be possible with PBSA encapsulated within CS-nio-aloe nanoparticles (Fig. 18a).^53^ Chemical sunscreens such as octyl methoxycinnamate (34, OMC) are frequently used in sunscreen cosmetics. On the other hand, hazards such as skin-photosensitive responses might arise from direct skin contact. An intriguing way to improve the photostability of filters is to encase ultraviolet filters in microcapsules. In order to create synergistic sunscreen microcapsules using sophisticated freezing technology, sodium caseinate (SC) and arabic gum (GA) were used as the wall materials. The impact of pH, wall substance concentration, and wall/core ratio in the development of OMC microcapsules has been studied through many studies. The OMC microcapsules' shape, composition, and stability are assessed using TGA, FTIR, and SEM. The OMC microcapsule exhibits a smooth surface shape, consistent size distribution, and strong heat stability. The findings demonstrate that, for UV-B (280–320 nm), the OMC microcapsules' absorption of UV is superior to that of the uncoated OMC. Furthermore, in twelve hours, the OMC microcapsule released 40% and OMC released 65%; nevertheless, the OMC microcapsule sunscreen has a sun protection factor that is 18.75% greater than OMC's. The hydrophobic connection between SC and OMC and the electrostatic bond between SC and GA may be responsible for this occurrence (Fig. 18b).^54^

**Fig. 19:**
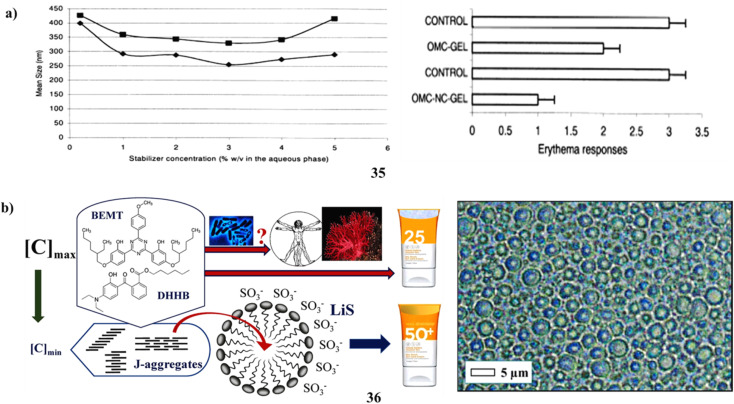
(a) Photoprotection of polymer nanocapsules (35) reproduced with permission from ref. 55 Copyright [2001] [Elsevier Publisher]. (b) Sunscreen formulations of lignosulfonate (36) reproduced with permission from ref. 56 Copyright [2021] [Elsevier Publisher].

Solvent displacement was used to manufacture biodegradable polymer nanocapsules (35) that contained the lipophilic sunscreen Parsol MCX (OMC) as the oil core. To look into the formulations' photoprotective potential, and OMC loading ability, with stabilizing agent effects (polysorbate 85, P-85, and poloxamer 188, P-188). The fast diffusion of the solvent across the contact is likely what causes the surface instability that leads to the creation of nanocapsules. It was determined that the stabilizing agents' capacity to prevent coalescence during solvent diffusion was what made them successful. P-85 outperformed P-188 as a poly(ε-caprolactone) nanocapsule stabilizer. OMC had a large loading capacity. The high lipophilicity of the medication and the hydrophobicity and crystallinity of the polymer control the *in vitro* release of OMC-nanocapsules. The OMC nanocapsules offer significantly better partial protection against UV-induced erythema when compared to a conventional gel, as illustrated in (Fig. 19a).^51^ Through an experimental design approach, Na-lignosulfonates (36) have been appropriately reevaluated as ideal pairings with two meticulously selected and well-explained safe filters. The demonstration indicates the potential to mitigate risks to the marine environment and human health by providing a range of photostable, non-cytotoxic emulsions spanning from SPF15 to SPF50, all available upon request. These emulsions contain only 5% LiS and minimal concentrations of organic filters. The absence of an ideal solar filter arises from the often unclear mechanisms governing microbial breakdown and accumulation in ecosystems. Prolonged exposure to the sun, even with a high SPF sunscreen, doesn't guarantee protection from serious skin damage. For instance, by adjusting the adsorption characteristics at the solid–liquid interface and optimizing the structure of micellar sunscreens, it might be possible to reduce the levels of BEMT and DHHB. As an additional starting point, 10% middle-size OLV lignin colloidal spheres have increased the sun protection factor (value of sunscreen with only 2% organic filters, from 10.7 to 47.7). The SPF values found *in vitro* in this study are accurate; however, because the anti-inflammatory properties of BEMT and DHHB need the measurement of SPF *in vivo* for marketing (packing) and legal purposes, the final assessment will be inflated (Fig. 19b).^56^

**Fig. 20:**
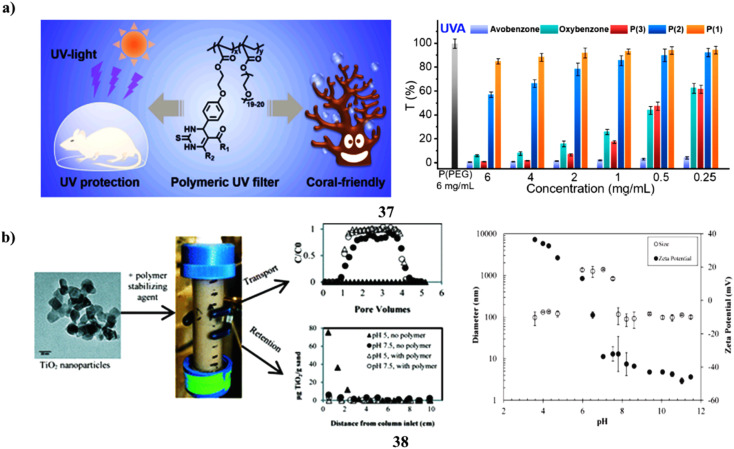
(a) UV protection of coral compounds (37) reproduced with permission from ref. 57 Copyright [2023] [Elsevier Publisher]. (b) Polymer sunscreen additive of titanium dioxide nanoparticles (38) reproduced with permission from ref. 58 Copyright [2016] [RSC Publisher].

The use of sunscreen is advised to shield human beings from UV rays that can cause harm and the onset of cancer. However, due to their small molecular weights, small-molecule organic UV filters included in sunscreens harm the ecosystem and could endanger users' health through transdermal action. An approach for combining the Biginelli reaction and free radical polymerization to create polymeric (37) UV screens that are secure and coral-friendly. They have created a polymer that is water-soluble with exceptional UV absorption that effectively shields mice from skin burns caused by UV radiation much better than renowned UV filters and over-the-counter sunscreens. Despite its high molecular weight, this polymer cannot be applied topically and is almost nontoxic to mice, algae, and corals. Understanding to create a bio- and coral-friendly polymeric UV filter using a straightforward multicomponent reaction will help in the creation of functional polymers with additional value for real-world applications (Fig. 20a).^57^ Paints, sunscreens, cosmetics, food, and other consumer goods all include titanium dioxide nanoparticles. Whenever dispersed into the environment, stabilizing substances found in these goods may change the fate of nTiO_2_. nTiO_2_ transport and deposition behavior in porous media as a result of the actions of TEGO carbomer (38), a polymeric stabilizing ingredient used in sunscreen. Columns filled with Federal Fine Ottawa sand were submerged in aqueous nTiO_2_ solutions at pH 5.0 or 7.5 ± 0.2. At pH 5, which is within the predicted point of zero charge (PZC) of nTiO_2_ (pH 6.3), nTiO_2_ was not found in effluent samples in the absence of carbomer, but more than 80% of nTiO_2_ was seen to elute at pH 7.5. The elution of nTiO_2_ was greater than 94% at pH 5 and 7.5 after the addition of 3 mg L^−1^ carbomer, which reduced the PZC from 6.3 to less than 5. The column breakthrough and retention data were captured using a nanoparticle transport model that included a first-order, maximal retention capacity term. According to model outcomes, regardless of changes in solution chemistry, the addition of carbomer decreased the typical solid phase retention capacity from 3.40 to 1.10 g TiO_2_ per g sand. These results show that polymeric stabilizing agents can significantly affect nTiO_2_ destiny in porous media, potentially increasing nTiO_2_ mobility in the surroundings and decreasing nTiO_2_ filtration system efficacy (Fig. 20b).^58^

**Fig. 21:**
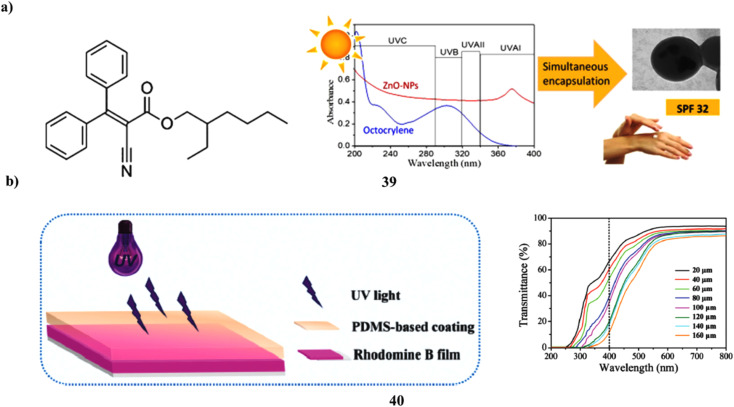
(a) Sunscreen formulations of octocrylene in poly (methyl methacrylate-*co*-styrene) nanoparticles (39) reproduced with permission from ref. 59 Copyright [2019] [Elsevier Publisher]. (b) Skin sunscreens based on polydimethylsiloxane coatings (40) reproduced with permission from ref. 60 Copyright [2020] [Elsevier Publisher].

Human skin fibroblasts are used to investigate the biocompatibility and sunscreen performance of a new sunscreen that simultaneously encapsulates zinc oxide nanoparticles and octocrylene in poly-styrene-*co*-methyl methacrylate (39, PMMA/PS) nanoparticles by the use of mini emulsion polymerization. PMMA/PS nanoparticles with excellent encapsulation efficiency and positive physical–chemical characteristics were effectively used to encapsulate both organic and inorganic filters for use in sunscreens. After being added to Artistoflex AVC gel, the nanoparticles produced a semi-solid formulation that was white, had a pH that was similar to the skin's pH and was homogenous in all respects. The semi-solid product with ZnO and octocrylene in PMMA nanoparticles demonstrated a good sun protection factor (SPF > 30), earning a 4-star rating from the Boots Star Rating System and being regarded as a good UVA sunscreen(Fig. 21a).^59^ Simply combining UV filters with aqueous cross-linkable PDMS coatings has allowed for the effective creation of PDMS-based skin sunscreens (40). Three kinds of sunscreens, PI, PO, and POI, were made using Mg/Al + Fe LDHs and an organic UV absorber, both in combination and independently. By using the hydrosilylation method at room temperature, all sunscreens can transform into transparent elastic films with good UV protection throughout the whole UV range (200–400 nm), skin analog mechanical performance, high WVT, and moderate adhesion strength. In the meantime, the films' WVT rate and mechanical strength may be improved by Mg/Al + Fe LDHs. However, especially at high UV, the organic UV absorber may reduce the mechanical strength and cause the PO films' surface to become greasy or even sticky. The oily feeling of PO films might be effectively eliminated by adding a tiny amount of Mg/Al + Fe LDHs. It's interesting to note that the POI sunscreen, which has 2.08 weight percent organic UV absorber and 0.69 weight percent Mg/Al + Fe LDHs, showed a sun-shielding performance that was on par with the high SPF commercial sunscreens. Such a modest UV filter concentration effectively mitigates sunscreen safety concerns (Fig. 21b).^60^

Polymeric nanoparticles in sunscreens offer enhanced stability and improved UV protection due to their ability to encapsulate UV filters, but there are concerns about their potential penetration into the skin and the environment, which requires further research and regulation to ensure their safety and environmental impact.

## 5. Metal oxide nanoparticles based sunscreen

**Fig. 22:**
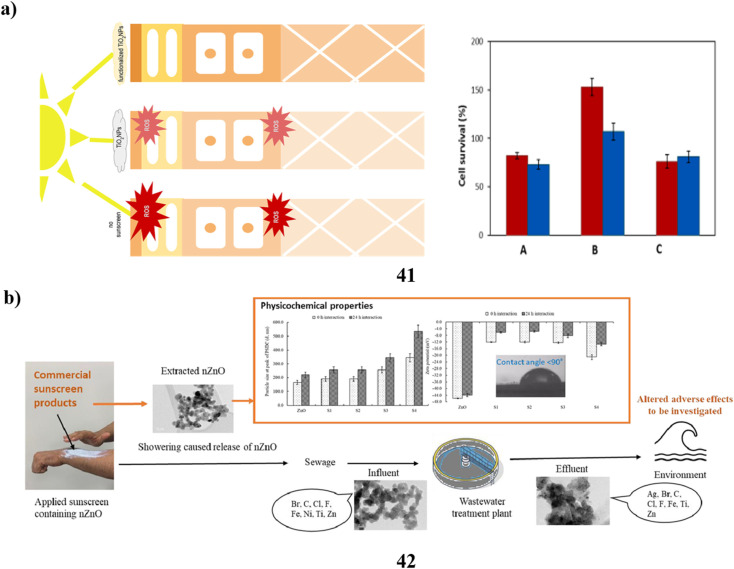
(a) *In vitro* study of TiO_2_ nanoparticle sunscreens (41) reproduced with permission from ref. 61 Copyright [2023] [Elsevier Publisher]. (b) Physicochemical study of ZnO nanoparticle sunscreens (42) reproduced with permission from ref. 62 Copyright [2023] [Elsevier Publisher].

In cosmetics, titanium dioxide nanoparticles (41) are often utilized. It's notably present in sunscreens because of its ability to absorb UV radiation. Their biocompatibility is still debatable, though. In particular, both *in vitro* and *in vivo* studies have been done on Degussa P25 (P25TiO_2_NPs) exposed to solar-simulated radiation. Following a 6 hours exposure to P25TiO_2_NPs and light the integrity of tissues and cell viability were impacted with TEM providing evidence of decreased tissue quality along with potent oxidative stress indicators. A novel biocompatible substitute based on the fast sol–gel functionalization of titanium dioxide nanoparticles with vitamin B2 has been developed to prevent these undesirable consequences. These nanoparticles with functional properties did not exhibit any of the phototoxicity effects (Fig. 22a).^61^ Additionally, the particles of a size determined in nanometers are present in sunblock based on zinc oxide (42). In this investigation, nanoparticles were found across four commercial sunscreens. The sunscreen-derived nanoparticles exhibited diverse morphologies, aspect ratios, and broad size ranges. When examining the size of the particles and charge on the surface of each nanoparticle, accumulation in their behavior was seen at different time intervals. Additionally, the characteristics of the nanoparticle content in the extraction and bought materials were compared with those of the genuine wastewater samples. According to the comparison, iron particles and co-contaminants with other organic components were found to be the second most common composition of nanoparticles found in wastewater samples, behind zinc, titanium, and silver elements. This experiment demonstrated the different morphologies of nanoparticles isolated from sunscreens and wastewater. Changes in the morphology, shape, and size of nanoparticles resulting from interactions with substances present in wastewater, seawater, or surface water have the potential to modify their negative effects on aquatic organisms. These impacts may differ from those observed when using pristine nanoparticles (Fig. 22b).^62^

**Fig. 23:**
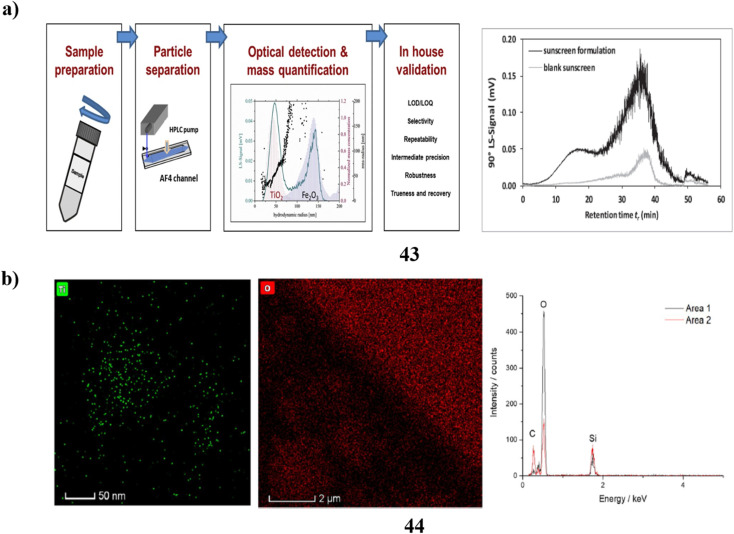
(a) Intra-laboratory assessment of TiO_2_ nanoparticle sunscreens (43) reproduced with permission from ref. 63 Copyright [2020] [Elsevier Publisher]. (b) Electron microscopy techniques of nanoparticle sunscreen (44) reproduced with permission from ref. 64 Copyright [2023] [Springer Publisher].

Additionally, an intra-laboratory evaluation was conducted to determine the efficacy of a technique for identifying TiO_2_-engineered nanoparticles (43) that are found in sunscreen that contains both upper nanometer-range and nanoscale TiO_2_ in addition to iron oxide particles. To produce the measurement errors related to the mass-based particle size distribution calculation with quantitative asymmetrical flow field-flow fractionation (AF4) calculation of the hydrodynamic radius, three duplicate measurements were conducted over five different days. The analysis of TiO_2_ ENPs found in sunscreen using AF4 separation-multi detection yields quantitative results with uncertainty based on the accuracy of 3.9–8.8%. As a result, this approach can be regarded as having excellent accuracy. Lastly, the bias data indicates that the lack of a sunscreen standard comprising certified TiO_2_ENPs means that the accuracy of the approach (*u*_t_ = 5.5–52%) can only be considered as a proxy (Fig. 23a).^63^ The most prevalent active components in plenty of commercial goods, including sunscreen, are nanoparticles (44). Therefore, it is essential to accurately characterize the nanoparticles present in these items to improve product design and comprehend the potential toxicological effects of the nanoparticles. While bulk methods may provide some helpful information, they frequently are unable to distinguish individual particles; as a result, high-resolution nanoparticle characterization is frequently achieved *via* electron microscopy. Still, unique *in situ* techniques must be employed because the traditional high vacuum dry TEM does not correctly portray nanoparticle dispersions. The researchers employ a range of methodologies, such as liquid cell transmission electron microscopy, cryogenic (cryo)-TEM, and cryoscanning electron microscopy to examine a commercial sunscreen that incorporates zinc oxide and titanium dioxide. Sample preparation is not necessary for LCTEM analysis since it can detect ZnO dissolution by the use of merely TiO_2_ nanoparticles due to beam artifacts. In contrast, ZnO and TiO_2_ may be characterized using cryo-TEM, but only pure products (without dilution) can be analyzed using cryo-SEM, which biases the characterization towards the higher proportion of agglomerates and nanoparticles. Ultimately, to ensure efficient and secure product design and production, a precise characterization of market items can only be done using a mix of several *in situ* EM methods (Fig. 23b).^64^

**Fig. 24:**
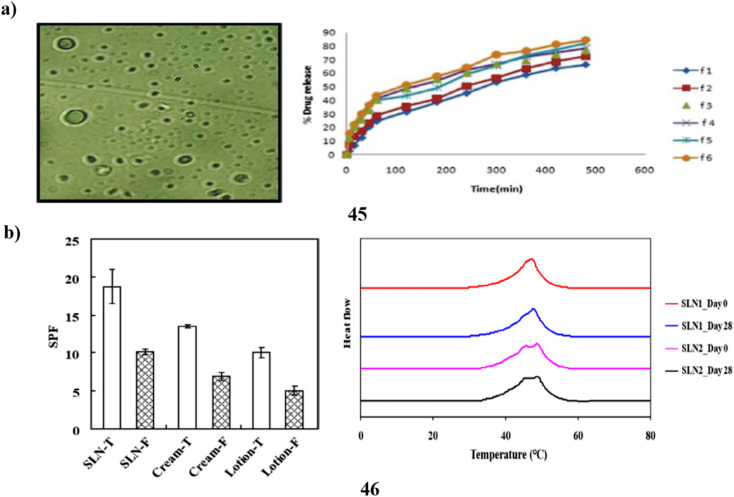
(a) Photoprotective potential of solid lipid nanoparticle-based sunscreen (45) reproduced with permission from ref. 65 Copyright [2020] [Springer Publisher]. (b) Sunscreen boosting effect of solid lipid nanoparticles (46) reproduced with permission from ref. 66 Copyright [2020] [MDPI Publisher].

A customized solvent emulsification approach was utilized to effectively produce the SLNs (45), and the resulting SLN dispersion was then added to a cream base for topical administration. To create the solid lipid nanoparticles of the photoprotective plant *Aloe vera*, glyceryl monostearate was used as the lipid and Tween 80 as the surfactant. The drug's release profile demonstrated enhanced topical withholding of *Aloe vera* for an extended amount of time. The lotion was found to have an outstanding factor for sun protection and increased photoprotective activity. The sunscreen formulation underwent a skin irritation test, and the results revealed no indications of hypersensitive reactions or discomfort. Because metallic compounds are removed from sunscreen formulations, the shielding effect of herbal nanoformulations opens the door for the future use of solid lipid nanoparticles of botanical powders and extracts in cream to obtain additional beneficial advantages with little toxicity (Fig. 24a).^65^ Fucoxanthin (46) is a naturally occurring carotenoid that is considered bioactive. Although fucoxanthin's polyunsaturated structure makes it physiochemically unstable to heat and acid, it is known for its protection against UV-B-induced cell destruction in hairless mice. This means that fucoxanthin has a low bioavailability, which restricts its use in the cosmetics industry. Systems of solid lipid nanoparticles are recognized for their suitability as sunscreen agent carriers. In this study, the sunburn protection factors of a macroemulsion as well as an SLN formula containing different types of sunscreen agents, respectively, were compared to assess the sunscreen-boosting effect of SLN as a carrier of functional ingredient, particularly fucoxanthin. Particle size, DSC analysis, X-ray analysis, stability test, and other results indicate the fact that SLN formula loading fucoxanthin may be a stable and highly effective ingredient delivery system. Additionally, compared to other formulas, the SLN recipe has demonstrated a greater SPF rating, indicating that it has a good sunscreen-boosting impact. The study suggests that the utilization of SLN as a carrier could improve the bioavailability of fucoxanthin and enable the manufacturing of sunscreen products at a larger scale (Fig. 24b).^66^

**Fig. 25:**
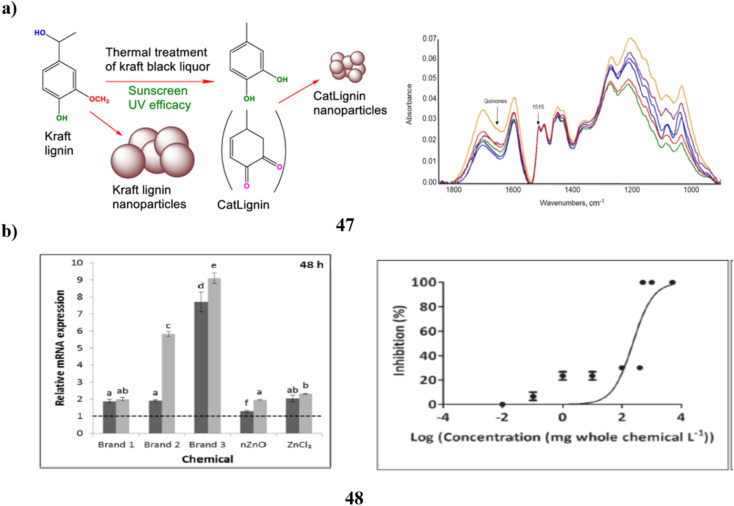
(a) Absorption study of sunscreens based on nanoparticles (47) reproduced with permission from ref. 67 Copyright [2020] [ACS Publisher]. (b) Toxicity study of sunscreens-based ZnO nanoparticles (48) reproduced with permission from ref. 68 Copyright [2020] [Elsevier Publisher].

The UV absorbers made of lignin were used to create safer bio-based sunscreens (47). Sunscreen UV absorbers with UVB-SPF and UVA–UVB transmittance values that are higher than those of conventional kraft lignins were the so-called CatLignins-partially demethylated and otherwise changed kraft lignins with an abundance of phenolic hydroxyl auxochromes and catechol units. Making lignins into nanoparticles greatly improved the performance of sunscreens. The UV transmittance of the best lignin sunscreen, which contained woody CatLignin nanoparticles, was just 0.5–3.8% throughout the UVA–UVB area, as opposed to 2.7–51.1% for a commercial SPF 15 sunscreen. Sunscreens based on lignin are especially good for applications with dark SPF tints (Fig. 25a).^67^ In simpler terms, the study looked at zinc oxide nanoparticles (48, nZnO) from three different sunscreens that can leach into human skin. The leaching rate varied among the sunscreens, ranging from 8% to 72%. They then tested the toxicity of these sunscreens on a tiny marine creature called *Tigriopus japonicus*. The results showed that the sunscreens had different levels of toxicity, with the concentration needed to cause harm varying. The three sunscreens' relative 96 hours median fatal concentrations for *T. japonicus* were determined to be >5000, 230.6, and 43.0 mg chem per L, which translates to Zn^2+^ concentrations of >82.5, 3.2, and 1.2 mg Zn per L. Based on the outcomes of *in vivo* tests, *T. japonicus* showed a rise in the expression of antioxidant genes and the generation of reactive oxygen species after being exposed to each sunscreen for ninety-six hours at ecologically realistic doses. It appears that marine life may be in danger from a state of oxidative stress caused by these sunscreens containing nZnO (Fig. 25b).^68^

**Fig. 26:**
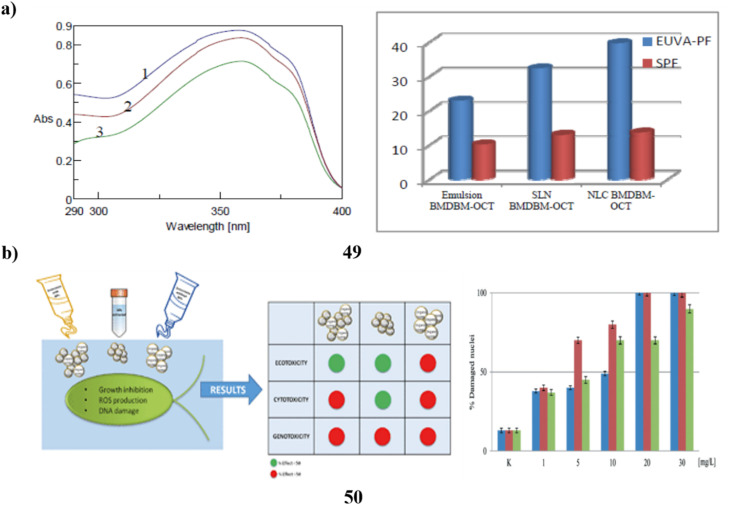
(a) Absorption study of lipid nanoparticles for sunscreen encapsulation (49) reproduced with permission from ref. 69 Copyright [2013] [ACS Publisher]. (b) Adverse effects of nanoparticles based sunscreens (50) reproduced with permission from ref. 70 Copyright [2018] [UPB Scientific Bulletin Publisher].

To prepare certain free lipid nanostructures appropriate for co-encapsulating UV-A and UV-B filters (BMDBM and OCT), the modified high-s hear homogenization method may be utilized. To co-encapsulate sunscreens, the SLNs (49) made with a 3.5% surfactant concentration using the tween 20/poloxamer/lecithin surfactant system and the NLCs made with the same surfactant mixture plus 3% liquid lipid were selected. The lipid nanoparticles in dispersing and the developed cosmetic formulations have better UV-blocking properties when sunscreens are added. The UV-blocking effectiveness of lipid matrix and organic UV filters is improved by the presence of liquid lipids in NLCs. It is possible to decrease the number of natural filters in the final cosmetic formulation without sacrificing photoprotection or adverse effects by enhancing the UV-blocking performance of sunscreens encapsulated into lipid nanoparticles (Fig. 26a).^69^ The harmful effects of NPs (50) in sunscreen compositions on *D. tertiolecta*, a marine microalgae. More significant toxicity was shown in sunscreens including NPs when zinc was also present in combination with TiO_2_ in the form of ZnO NPs and Zn stearate. This implies that zinc exerts toxicity in sunscreen formulations to a considerable extent. The primary indicators of the impact were the generation of ROS and DNA damage. As a result, the algae were exposed to NPs isolated from the sunscreens, which had genotoxic effects. Growth suppression was also seen in the sunscreens containing ZnO NPs. The reactions that the NPs isolated from sunscreens displayed were not consistent with those seen in research using industrial nanoparticles. This suggests that the aging process of sunscreens may have an impact on the ultimate toxicity of TiO_2_ nanoparticles. When comparing the outcomes of tests conducted with sunscreens and their corresponding nanoparticle extracts, it became evident that the overall sunscreen products induced more pronounced adverse effects. This suggests that components in the sunscreen formulations, aside from nanoparticles, might possess toxic properties. This observation is supported by the toxicity exhibited by sunscreens formulated without nanoparticles. Additionally, there's a possibility that these components may act in synergy with nanoparticles, amplifying their overall impact on biological systems (Fig. 26b).^70^

**Fig. 27:**
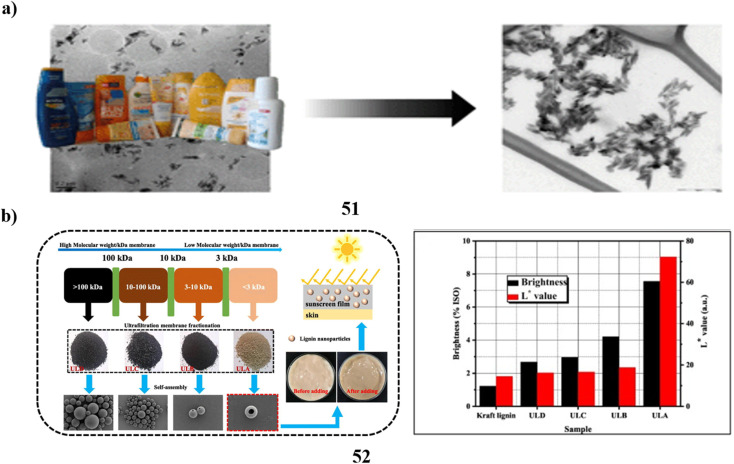
(a) Morphology study of TiO_2_ nanoparticle sunscreens (51) Reproduced with permission from ref. 71 Copyright [2018] [RSC Publisher]. (b) UV-blocking sunscreen-based lignin nanoparticles (52) reproduced with permission from ref. 72 Copyright [2023] [Elsevier Publisher].

TiO_2_ nanoparticles (51) in surface waters are mostly sourced from sunscreens. Due to the difference between model nanoparticles often employed in studies and the more complex particles seen in commercial products, the fate and toxicities of these particles have not been completely explored. To offer more realistic nanoparticle samples for the next research, moderate extraction techniques for TiO_2_ nanoparticles using sunscreens were examined. Two techniques for removing TiO_2_ nanoparticles from sunscreens that use a surfactant solution as the solvent are based on ultrafiltration as well as ultracentrifugation, respectively. Eleven different commercial sunscreens with varying compositions were used to test these strategies. About 5 g of sunscreen can have 250 mg extracted from it in a single day using the ultracentrifugation form. The recovery rates for ultrafiltration and ultracentrifugation were 52–96% and 78–98%, respectively. By employing UV spectrometry to measure the amount of avobenzone in sunscreen extracts, the ultracentrifugation variant's purification effectiveness was found to be high across all tested sunscreens. While size characteristics were similar a substantial degree of variability in particle shape was found using a combination of transmission electron microscopy and dynamic light scattering. The isoelectric points of every sunscreen extract were less than 4.6. All TiO_2_ particles were most likely coated, with the majority of them being coated with PDMS and the remaining ones with materials based on Al and Si, according to time-off light secondary ion mass spectrometry. While the geometry of the main nanoparticles was unaffected by the extraction process, they were aggregated inside the sunscreens, according to a comparison of images acquired through cryogenic transmission electron microscopy of the particles inside the sunscreens and removed particles. Ultrasonication could break apart these agglomerates. As a result, compared to model TiO_2_ nanoparticles the extracted particles' size, shape, surface charge, and coating might be thought of as more globally significant (Fig. 27a).^71^ Kraft lignin (52, KL) can be used in a variety of ways. However, KL is difficult to employ in skincare and nanoparticle manufacturing due to its dark color and large size distribution. Using the ultrafiltration membrane fractionation, the paper-making company separated KL, yielding four distinct types of lignin with varying molecular weights. After that, four different varieties of UL were used to self-assemble to create lignin nanoparticles or ULNPs. Low molecular weight lignin, such as ULA, showed good antioxidant qualities (89.47%, 5 mg mL^−1^), a high brightness (ISO% = 7.55), high L* value (L* = 72.3), and low polydispersity index (PDI = 1.41), according to an analysis of the UL and ULNP properties. The ULNP exhibited a high dispersion in sunscreen and a restricted size distribution (0.8–1.4 m). Sunscreen's sun protection factor value surged from 14.93 to 63.74 when ULNP was applied at a 5% load. Consequently, this study provided a useful method for the full utilization of pulping waste KL (Fig. 27b).^72^

**Fig. 28:**
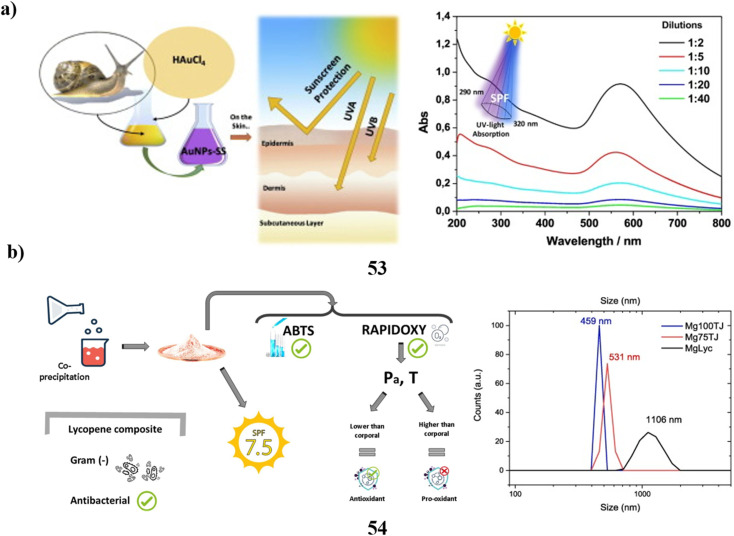
(a) Absorption spectra of snail slime-based gold nanoparticles (53) reproduced with permission from ref. 73 Copyright [2021] [Elsevier Publisher]. (b) Absorption spectra of double hydroxide nanoparticles (54) reproduced with permission from ref. 74 Copyright [2023] [Elsevier Publisher].

Products made from snail slime are well-liked and utilized all over the world. As a result, gold nanoparticles were created using snail slime as a novel and alternative method, giving them intriguing features. The major components of the slime were used to design the inorganic metallic core of the 14 ± 6 nm wide hybrid gold nanoparticles (53), which were created using a straightforward, one-pot method. Among their other characteristics, different antioxidant and tyrosinase inhibitory activities were investigated using the DPPH and ABTS and tyrosinase tests, respectively. Positive outcomes allowed for their usage as an intriguing novel multifunctional cosmetic component. However, the photostability of gold nanoparticles, which was studied using a solar simulator lamp, points to their possible usage as a substitute for the inorganic sunscreen components that are often found in conventional cosmetic sunscreen solutions. The presumed Sun Protection Factor was assessed, and values between 0 and 12 were obtained. The research of gold nanoparticles derived from snail slime as a possible multipurpose platform in cosmetics has never been more appealing thanks to the suggested ecologically benign and economically advantageous nanoparticle production methodology, which adheres to the principles of Green Chemistry (Fig. 28a).^73^ The Layered double hydroxides (54, LDH) are adaptable building blocks for creating cutting-edge materials. Rarely is the necessity to investigate green or sustainable approaches mentioned. In this study, the sun protection factor and antioxidant qualities of LDH composites made with tomato-derived natural ingredients. According to the findings, the composite materials' 11% organic matter concentration is enough to boost their antioxidant capabilities, such as their stronger antioxidant activity towards ABTS^+^ than towards DPPH. The composite additionally reduces the amount of oxygen atmospheric degradation that the Rapidoxy assay could detect, while the SPF revealed that the LDH particles, rather than the organic content are more important for sunscreen protection. The composite of lycopene and LDH particles increases lycopene's hydro-dispersibility and boosts its antioxidant stability, both of which are crucial characteristics for creating cosmetic or dietary components (Fig. 28b).^74^

**Fig. 29:**
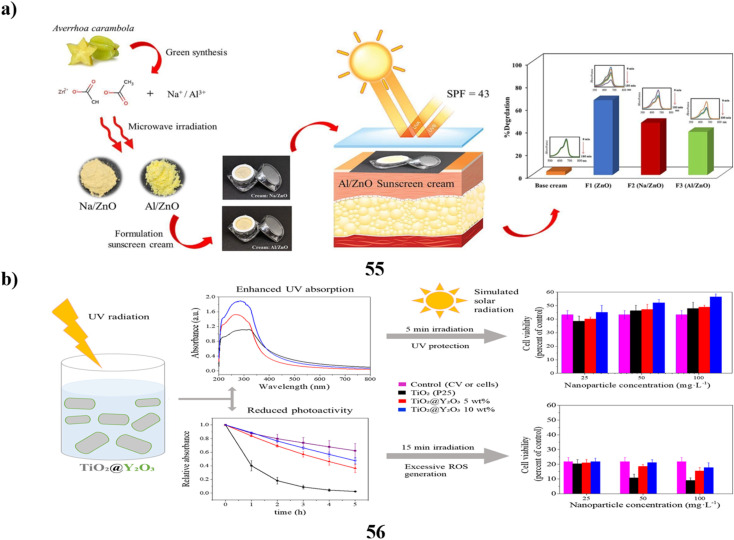
(a) Potential application of sunscreens based Na and Al doping on ZnO nanoparticles (55) reproduced with permission from ref. 75 Copyright [2023] [Elsevier Publisher]. (b) Photocatalytic activity of Y_2_O_3_ decorated TiO_2_ nanoparticles (56) reproduced with permission from ref. 76 Copyright [2020] [Elsevier Publisher].

In addition, zinc oxide nanoparticles (55) doped with the elements Al & Na metals are produced and characterized in a way that is environmentally conscious to reduce the photocatalytic function of ZnO for use in sunscreen. The reducing agent for the metal-doped zinc oxide materials was extracted from *Averrhoa carambola*, popularly known as star fruit, and manufactured utilizing the microwave process. Using techniques such as TEM, EDX, SEM, UV-vis spectroscopy, and XRD the effects of metal-ion doping on the crystalline structure, morphology, and optical properties of ZnO were examined. The sunscreen formulations with undoped ZnO, Na-doped ZnO, and Al-doped ZnO NPs had sun protection factors of 10.10, 25.10, and 43.08, respectively. As a result, the SPF of Na/ZnO and Al/ZnO was higher. Furthermore, the produced sunscreens and nanomaterials showed antioxidant properties and were efficient against a variety of bacteria, including Gram-positive as well as Gram-negative. The photocatalytic activity of the undoped ZnO, Na/ZnO, and Al/ZnO NPs were assessed using the methylene blue (MB) degradation method. The results showed that the rates were 66%, 46%, and 38%, respectively. Consequently, ZnO NPs' photocatalytic activity was reduced with Na- and Al-doping because of their structural flaws. Al/ZnO is also a prime choice for a component in sunscreens(Fig. 29a).^75^ Hydrothermal synthesis was used to create TiO_2_@Y_2_O_3_ nanoparticles (56) with Y/Ti weight ratios of 5 and 10 wt%. When Y_2_O_3_ was added to TiO_2_, it was discovered that, in comparison to pristine TiO_2_ (P25), there was less scattering in the visible region and more absorbance in the UVB and short UVA wavelength ranges. Additionally, under both UV and simulated solar irradiation, these composites significantly reduced the photoactivity of TiO_2_ (P25), as measured by dye staining. The fundamental process behind this decrease was attributed to Y_2_O_3_'s inhibition of free radical production and active charge carrier transfer blockage from the coated layer. Under all test settings, the composite particles showed the greatest HaCaT cell vitality with cell viability rising with surface Y_2_O_3_ loading. The composites seemed to support cell survival and proliferation in a concentration- and yttria loading-dependent way when UV light was absent. It is hypothesized that this results from the less active surface-treated particles inducing less oxidative stress. TiO_2_-based nanoparticles were demonstrated to produce opposing effects under simulated solar radiation, with increased cell death and protection against exposure to radiation at low and high radiation levels, respectively. The established UV absorption and photocatalytic efficiency of the examined materials, respectively, were ascribed to these biological reactions. Therefore, TiO_2_@Y_2_O_3_ 10 wt% showed the best protection and least amount of induced cell death, followed by TiO_2_ (P25) and the 5 wt% composite. The yttria-based nanocomposites exhibit enhanced optical characteristics and biocompatibility, along with a decrease in photocatalytic activity. These findings underscore the possible advantages of incorporating these materials into sunscreen formulations (Fig. 29b).^76^

**Fig. 30:**
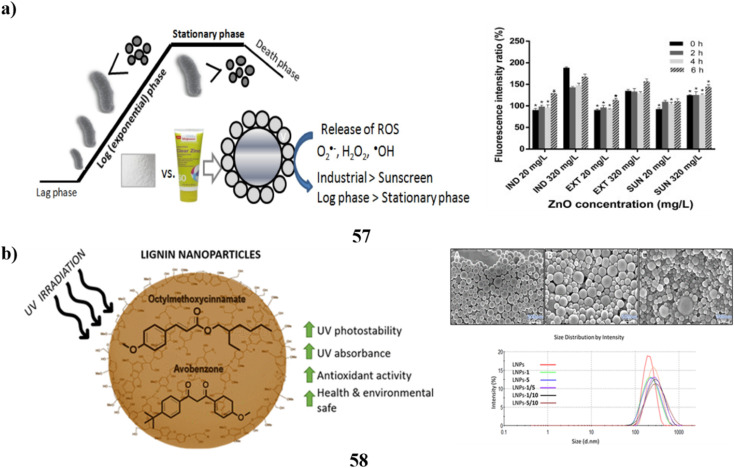
(a) Antibacterial effect of sunscreen-based ZnO nanoparticles (57) reproduced with permission from ref. 77 Copyright [2017] [Elsevier Publisher]. (b) Photoprotective study of lignin nanoparticles (58) reproduced with permission from ref. 78 Copyright [2022] [ACS Publisher].

The nanoparticles are pushing the boundaries in science, technology, medicine, and consumer products, concerns are growing about their potential toxicity to human health and the environment. To assess the toxicity of zinc oxide nanoparticles ZnO NPs (57), researchers compared sunscreen-extracted ZnO NPs with industrial-grade ZnO NPs. They exposed *E. coli* bacteria to varying concentrations of ZnO NPs for different durations. The analysis revealed that the growth of *E. coli* and the production of reactive oxygen species (ROS) depended on factors such as particle type, size, and the level and duration of exposure to ZnO NPs. ROS generation was observed to be higher during the growth phase compared to the stationary phase. Notably, industrial ZnO NPs with smaller particle sizes exhibited greater toxicity than those extracted from sunscreen, which had larger particle sizes. The high toxicity of smaller particles with a homogeneous distribution is likely attributed to their size, suggesting a potential underlying cause for their increased harmful effects (Fig. 30a).^77^ Additionally, lignin nanoparticles (58) function as photostabilizers and sustainable carriers of two widely used UV chemical filters: octyl methoxycinnamate and avobenzone. By employing deionized water as an antisolvent and eco-certified dimethyl the isosorbide for the principal solvent, the chemicals were encapsulated into kraft lignin NPs by nanoprecipitation. Both compounds greatly increased the half-life stability against UV irradiation after encapsulation. Coencapsulating avobenzone and octyl methoxycinnamate with hydroxytyrosol a naturally occurring phenol with antioxidant activity that was recovered from olive oil wastes and was known for its skin-regenerating qualities improved the stabilizing qualities of lignin nanoparticles even further (Fig. 30b).^78^

**Fig. 31:**
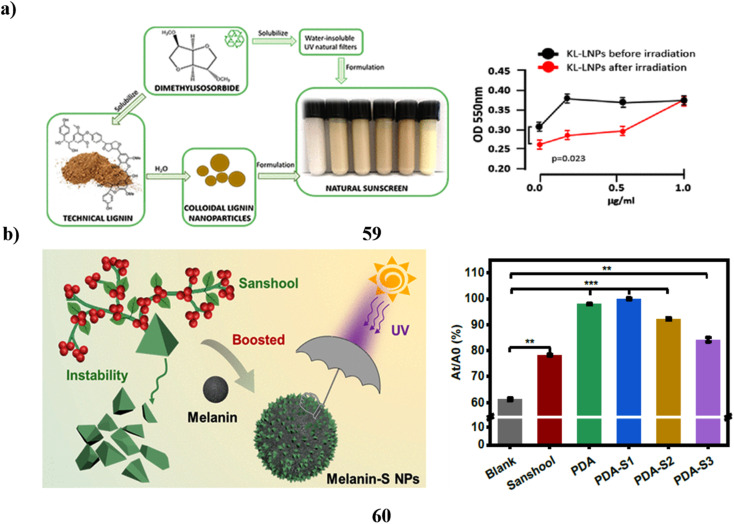
(a) Sunscreen formulations of lignin nanoparticles (59) reproduced with permission from ref. 79 Copyright [2021] [ACS Publisher]. (b) Photoprotective efficiency of melanin-like nanoparticles (60) reproduced with permission from ref. 80 Copyright [2023] [ACS Publisher].

Lignin nanoparticles (LNPs) (59) are used in a variety of industrial settings. Although LNPs may be nanoprecipitated quickly and affordably, the process still requires the use of organic solvents that might be dangerous, which makes their widespread application challenging. A scalable method of nanoprecipitation using isopropylidene glycerol and dimethylisosorbide, two environmentally friendly chemical solvents, to produce colloidal lignin nanoparticles (cLNPs). Compared to parent LNPs and bare lignin, cLNPs demonstrated superior UV-absorbing qualities and antioxidant activity, regardless of the experimental setup. Following their application, cLNPs were utilized to create environmentally friendly sunscreen formulations that demonstrated strong UV-shielding activity even in the absence of physical filters (ZnO and TiO_2_) and artificial boosters (microplastics). Human HaCaT keratinocytes and human cutaneous equivalents were used in biological experiments to show that there was no cytotoxicity or genotoxicity, which is indicative of the best possible defense of the skin against UV-A damage (Fig. 31a).^79^ A naturally occurring compound derived from *Zanthoxylum xanthoxylum* is called Sanshool. It plays a crucial role in avoiding photodamage. However, its continued use is restricted by its innate instability and possible skin penetration danger. They devised a method using melanin-like materials to enhance the efficiency and stability of sanshool, resulting in the creation of melanin-sanshool nanoparticles (60). Through boron esterification interactions, the researchers successfully produced melanin-S NPs with consistent sizes, improved stability, effective ultraviolet absorption, and antioxidative capacities in laboratory settings. They then delved into studying the skin permeation, photoprotective activity, and potential mechanisms of melanin-S NPs using both cellular and animal models for skin photodamage. This bioinspired approach of utilizing melanin-based nanomaterials serves as a promising platform for enhancing the crucial properties of naturally occurring functional molecules like sanshool, opening up new possibilities for advanced photoprotective applications (Fig. 31b).^80,81^

Metal oxide nanoparticles-based sunscreens offer effective protection against both UVA and UVB rays while being less likely to cause skin irritation compared to chemical sunscreens. However, concerns have been raised about the potential environmental impact of metal oxide nanoparticles, particularly in marine ecosystems, and their potential to cause oxidative stress in cells.

## 6. Conclusion

The development and application of Chromophore Compounds and Nanoparticles in sunscreens represent a significant stride in enhancing sun protection efficacy, photostability, and environmental sustainability. Each type of sunscreen has its own set of advantages and challenges, and the best choice often comes down to a balance between efficacy, safety, and cosmetic attributes. Metal oxide nanoparticles-based sunscreens, specifically those containing titanium dioxide and zinc oxide, are commonly preferred due to their proven broad-spectrum protection, reduced skin irritation, and improved cosmetic acceptability. They form a reliable and well-established choice for many individuals, particularly those with sensitive skin. Flavonoid and polymeric nanoparticle-based sunscreens show promise and may offer additional benefits, such as antioxidant properties and enhanced stability. However, these formulations are still undergoing research and development to optimize their effectiveness, address stability issues, and ensure safety for widespread use. If proven broad-spectrum protection, reduced skin irritation, and cosmetic acceptability are top priorities, metal oxide nanoparticle-based sunscreens may be the preferred choice. The incorporation of nanoparticles, notably zinc oxide and titanium dioxide, has revolutionized sunscreen formulations by offering broad-spectrum UV protection while maintaining an aesthetically pleasing appearance. Their ability to scatter and absorb UV radiation without leaving an unsightly residue on the skin is a pivotal advancement in the industry. Furthermore, the encapsulation of chromophore compounds within nanoparticles has shown promise in augmenting UV protection by selectively absorbing specific wavelengths of light. Advancements in photostability have resulted in sunscreens that endure longer periods of sun exposure, ensuring that the skin remains safeguarded throughout outdoor activities. The inclusion of antioxidants like vitamins C and E further fortifies the protective qualities of sunscreens, helping to neutralize the harmful free radicals generated by UV radiation. Sustainability has also been a central theme in recent research. The pursuit of eco-friendly, biodegradable sunscreen ingredients and formulations has gained momentum, in response to concerns regarding the environmental impact of sunscreen chemicals, particularly on coral reefs. Inclusivity in sun protection has been another focal point, with the development of sunscreens suitable for a wide range of skin tones, thereby addressing the diverse needs of the population. There isn't a universally agreed “best” sunscreen among benzophenone-based, nanoparticles-based, polymer-based, and flavonoid-based sunscreens. Each type has pros and cons. Benzophenone offers broad-spectrum UV protection but has hormone-disrupting concerns. Nanoparticles provide effective UV protection but have environmental and safety concerns. Polymer-based sunscreens are water-resistant but need more research on long-term effects. Flavonoid-based sunscreens have antioxidant properties but need more study on their stability. Research is focused on improving safety, efficacy, and environmental impact. When choosing, consider your skin type, conditions, and ethical concerns, and consult a professional for guidance. These innovations aim to offer more effective, environmentally responsible, and inclusive solutions for shielding the skin from the detrimental effects of UV radiation. Ultimately, the collaborative efforts of scientists, researchers, and the skincare industry contribute to a brighter future, where sunscreens become not only protective shields against the sun but also symbols of sustainable and inclusive care for our skin and the environment.

## Abbreviations

SSPs: Sunscreen Products
TPC: Total Phenol Content
TFC: Total Flavonoid Content
CNSL: Cashew Nutshell Liquid
SPF: Sun Protection Factor
UV: Ultraviolet
TiO_2_: Titanium Dioxide
ZnO: Zinc Oxide
PXRD: Powder X-ray Diffraction
TGA: Thermogravimetric Analysis
DTA: Differential Thermal Analysis
BPs: Benzophenones
DO: Dissolved Oxygen
ROS: Reactive Oxygen Species
LC-Q-TOF-MS: Time-of-Flight Mass Spectrometry
DFT: Density Functional Theory
DOB-PUL: Dioxybenzone-Pullulan Polymer
DOB: Dioxybenzone
NMR: Nuclear Magnetic Resonance
SET: Single Electron Transfer
HOMO: Highest Occupied Molecular Orbital
LUMO: Least Unoccupied Molecular Orbital
ESIPT: Excited State Intramolecular Proton Transfer
TDDFT: Time-Dependent Density Functional Theory
HDF: Human Dermal Fibroblasts
DNA: Deoxyribonucleic acid
SLN: Solid Lipid Nanoparticles
PCL: Poly(Epsilon-Caprolactone)
PBSA: Phenylbenzimidazole-5-Sulfonic Acid
FTIR: Fourier Transform Infrared
SEM: Scanning Electron Microscopy
TEM: Transmission Electron Microscopy
DLS: Dynamic Light Scattering
NPs: Nanoparticles
EDX: Energy Dispersive X-ray Spectroscopy
ROS: Reactive Oxygen Species
LNPs: Lignin Nanoparticles

## Conflicts of interest

Authors have no conflict of interest in any part of this manuscript.

## Supplementary Material

## References

[cit1] Resende D. I. S. P., Jesus A., Lobo J. M. S., Sousa E., Cruz M. T., Cidade H., Almeida I. F. (2023). Up-to-Date Overview of the Use of Natural Ingredients in Sunscreens. Pharmaceuticals.

[cit2] Cooney J., Lenczewski M., Bautista R. M. L., Tucker K., Davis M., Rodriguez J. (2023). Analysis of sunscreens and antibiotics in groundwater during the Covid-19 pandemic in the Riviera Maya, Mexico. Sci. Total Environ..

[cit3] Geoffrey K., Mwangi A. N., Maru S. M. (2019). Sunscreen products: Rationale for use, formulation development and regulatory considerations. Saudi Pharm. J..

[cit4] Fedunik L. (2023). Super sunscreen. New Sci..

[cit5] Kakuda L., Maia Campos P. M. B. G., Zanin R. B., Favaro L. N. (2023). Development of multifunctional sunscreens: Evaluation of physico-mechanical and film-forming properties. Int. J. Pharm..

[cit6] Cowden M., Whittock A. L., Holt E. L., Stavros V. G., Wills M. (2023). Synthesis and characterisation of novel composite sunscreens containing both avobenzone and octocrylene motifs. RSC Adv..

[cit7] Márquez M. G. G., Castañeda J. C. R., Agawin N. S. R. (2023). Sunscreen exposure interferes with physiological processes while inducing oxidative stress in seagrass Posidonia oceanica (L.) Delile. Mar. Pollut. Bull..

[cit8] Gebara M., Green M., Maibach H. (2023). Sunscreen decontamination: a call to action for further research. Arch. Dermatol. Res..

[cit9] Alsaidan M. S., Alsohaimi A., Alanazi Z. G., Alnefea A. Z., Alanazi R. M., Algraene T. S. (2023). Current practice and beliefs of parents toward sunscreen use for their children: A cross-sectional study. Prev. Med. Rep..

[cit10] Sharma M., Sharma A. (2023). A Review on Nature Based Sunscreen Agents. IOP Conf. Ser.: Earth Environ. Sci..

[cit11] Liu P., Guo Y., Guo G., Dai L., Hu G., Xie H. (2023). Lignin-grafting alternative copolymer of 3,4-dihydrocoumarin and epoxides as an active and flexible ingredient in sunscreen. Green Chem..

[cit12] Porrawatkul P., Nuengmatcha P., Kuyyogsuy A., Pimsen R., Rattanaburi P. (2023). Effect of Na and Al doping on ZnO nanoparticles for potential application in sunscreens. J. Photochem..

[cit13] Tong Q., Xiao Y., Yi Z., Chen X., Jiang X., Li X. (2023). Polyphenolic condensation assembly enabled biocompatible, antioxidative, and light-colored tea sunscreen formulations with broadband UV protection. Green Chem..

[cit14] Martins R. M., Martins S. S., Barbosa G. L. F., Silva E. G. N., Fonseca M. J. V., Freitas L. A. P. (2023). Natural component and solid lipid microparticles of solar filter in sunscreen: Photoprotective and photostability effect enhancement. J. Drug Delivery Sci. Technol..

[cit15] Righi S., Prato E., Magnani G., Lama V., Biandolino F., Parlapiano I., Carella F., Iafisco M., Adamiano A. (2023). Calcium phosphates from fish bones in sunscreen: An LCA and toxicity study of an emerging material for circular economy. Sci. Total Environ..

[cit16] Soleimani S., Yousefzadi M., Nezhad S. B. M., Pozharitskaya O. N., Shikov A. N. (2023). Potential of the Ethyl Acetate Fraction of Padina boergesenii as a Natural UV Filter in Sunscreen Cream Formulation. Life.

[cit17] Hong S., Peng Z., Wu M., Nie Y., Yi Y., Cai H., Zhang X.-Z. (2023). Human-Hair-Derived Natural
Particles as Multifunctional Sunscreen for Effective UV Protection. ACS Nano.

[cit18] Tugrul B., Demirdag H. G., Sahin A. H. (2023). Vitamin D Levels in Children During Winter and the Relationship Between Sunscreen and Sun Protection Behaviors. Dermatol. Pract. Concept..

[cit19] Chavda V. P., Acharya D., Hala V., Daware S., Vora L. K. (2023). Sunscreens: A comprehensive review with the application of nanotechnology. J. Drug Delivery Sci. Technol..

[cit20] Guo Y., Wang M., Wu Z., Shi Y., Wang Y., Zhang S., Jin B., Cui S., Zhao G. (2023). Ultrafast non-adiabatic dynamics of stilbene-based plant-derived sunscreens with cis-trans isomerization structures. Spectrochim.

[cit21] Cursino C. T., Lisboa F. S., Pyrrho A. S., Sousa V. P., Wypych F. (2013). Layered double hydroxides intercalated with anionic surfactants/benzophenone as potential materials for sunscreens. J. Colloid Interface Sci..

[cit22] Ge J., Huang D., Han Z., Wang X., Wang X., Wang Z. (2019). Photochemical behavior of benzophenone sunscreens induced by nitrate in aquatic environments. Water Res..

[cit23] Heo S., Hwang H. S., Jeong Y., Na K. (2018). Skin protection efficacy from UV irradiation and skin penetration property of polysaccharide-benzophenone conjugates as a sunscreen agent. Carbohydr. Polym..

[cit24] Li Y., Qiao X., Zhou C., Zhang Y., Fu Z., Chen J. (2016). Photochemical transformation of sunscreen agent benzophenone-3 and its metabolite in surface freshwater and seawater. Chemosphere.

[cit25] Manasfi T., Méo M., Coulomb B., Giorgio C., Ravier S., Jean-Luc B. (2019). Development of transient mutagenic activity following the chlorination of the sunscreen UV filter dioxybenzone (benzophenone-8) in bromide-rich water. Int. J. Hyg. Environ. Health.

[cit26] Downs C. A., DiNardo J. C., Stien D., Rodrigues A. M. S., Lebaron P. (2021). Benzophenone Accumulates over Time from the Degradation of Octocrylene in Commercial Sunscreen Products. Chem. Res. Toxicol..

[cit27] Zhan T., Cui S., Liu X., Zhang C., Huang Y. M., Zhuang S. (2021). Enhanced Disrupting Effect of Benzophenone-1 Chlorination Byproducts to the Androgen Receptor: Cell-Based Assays and Gaussian Accelerated Molecular Dynamics Simulations. Chem. Res. Toxicol..

[cit28] Shin J. C., Lee E., Ana S., Jin S. H., Ha J., Choi W. J., Noh M. (2020). Benzophenone-3 and benzophenone-8 exhibit obesogenic activity via peroxisome proliferator-activated receptor γ pathway. Toxicol. In Vitro.

[cit29] Li Z. M., Kannan K. (2022). Comprehensive Survey of 14 Benzophenone UV Filters in Sunscreen Products Marketed in the United States: Implications for Human Exposure. Environ. Sci. Technol..

[cit30] Nska A. W. S., KaBwbasiak K., Pyzowski J., BrzeziNska E. (2015). Quantification of Sunscreen Benzophenone-4 in Hair Shampoos by Hydrophilic Interactions Thin-Layer Chromatography/Densitometry or Derivative UV Spectrophotometry. J. Anal. Methods Chem..

[cit31] Castro A. P., Ruela A. L. M., Amaral J. G., Santos M. H., Pereira G. R., Marques M. J. (2019). A stability-indicating method by LC-UV for analysis of 7-epi-clusianone extracted from Garcinia brasiliensis fruits and a novel prenylated benzophenone from the oxidation of this molecular marker. Anal. Methods.

[cit32] Zhang B., Ruan J., Xie L., Gui M., Bai X., Zhang T. (2018). Urinary benzophenone-type UV filters in people living in South China: rural versus urban areas. Environ. Sci.: Processes Impacts.

[cit33] Cai H., Xia L., Lee Y. R. (2016). Regioselective construction of diverse and multifunctionalized 2-hydroxybenzophenones for sun protection by indium(III)-catalyzed benzannulation. Chem. Commun..

[cit34] Huang X., Wang X., Wang S., Yang J., Zhong L., Pan J. (2013). UV and dark-triggered repetitive release and encapsulation of benzophenone-3 from biocompatible ZnO nanoparticles potential for skin protection. Nanoscale.

[cit35] Akhtar M. S., Thombal R. S., Tamargo R. J. I., Yang W. G., Kim S. H., Lee Y. R. (2020). Eco-friendly organocatalyst- and reagent-controlled selective construction of diverse and multifunctionalized 2-hydroxybenzophenone frameworks for potent UV-A/B filters by cascade benzannulation. Green Chem..

[cit36] Rascón A. J., Bautista P. R., Colon L. P., Ballesteros E. (2023). Easy determination of benzophenone and its derivatives in sunscreen samples by Direct-Immersion Solid-Phase Microextraction and Gas Chromatography-Mass Spectrometry. J. Pharm. Biomed. Anal..

[cit37] Zamoiski R. D., Cahoon E. K., Freedman D. M., Linet M. S. (2015). Self-reported sunscreen use and urinary benzophenone-3 concentrations in the United States: NHANES 2003–2006 and 2009–2012. Environ. Res..

[cit38] Berbicz F., Nogueira A. C., Neto A. M., Natali M. R. M., Baesso M. L., Matioli G. (2011). Use of photoacoustic spectroscopy in the characterization of inclusion complexes of benzophenone-3-hydroxypropyl-β-cyclodextrin and ex vivo evaluation of the percutaneous penetration of sunscreen. Eur. J. Pharm. Biopharm..

[cit39] Cefali L. C., Ataide J. A., Fernandes A. R., Sousa I. M. O., Gonçalves F. C. S., Eberlin S., Dávila J. L., Jozala A. F., Chaud M. V., Sanchez-Lopez E. (2019). Flavonoid-Enriched Plant-Extract-Loaded Emulsion: A Novel Phytocosmetic Sunscreen Formulation with Antioxidant Properties. Antioxidants.

[cit40] Dianursanti S. T., Prakasa M. B., Nugroho P. (2020). The effect of adding microalgae extract Spirulina platensis containing flavonoid in the formation of Sunscreen towards cream stability and SPF values. AIP Conf. Proc..

[cit41] Zombe K., Nyirenda J., Lumai A., Phiri H. (2022). Impact of Solvent Type on Total Phenol and Flavonoid Content and Sun Protection Factor of Crude Cashew Nutshell Liquid. Sustainable Chem..

[cit42] Chinh N. T., Anh N. T. L., Thao P. T., Quang D. D. (2020). Photoprotective Properties of Natural Antioxidant Flavonoids: A DFT and TD-DFT study on Acridone Derivatives. Vietnam J. Chem..

[cit43] Badea N., Giurginca M., Meghea A. (2008). Complex Effects of Sunscreen Agents and Flavonoid Antioxidants Devoted to Enhance Photoprotection of Dermal Tissues. Mol. Cryst. Liq. Cryst..

[cit44] Utami R., Syahputra R., Dona R., Fadhli H., Furi M., Ikhtiarudin I. (2023). Total flavonoid content and in vitro study on the sunscreen activity of extracts of leaves of Elaeocarpus floribundus blume. Pharm. Educ..

[cit45] Hashemi Z. (2019). Sun protection factor, total phenol, flavonoid contents and antioxidant activity of medicinal plants from Iran. Trop. J. Pharm. Res..

[cit46] Polonini H. C., Brandao M. A. F., Raposo N. R. B. (2014). A natural broad-spectrum sunscreen formulated from the dried extract of Brazilian Lippia sericea as a single UV filter. RSC Adv..

[cit47] Barnes P. W., Tobler M. A., Ring K. K., Flint S. D., Barkley A. E., Ryel R. J., Lindroth R. L. (2016). Rapid modulation of ultraviolet shielding in plants is influenced by solar ultraviolet radiation and linked to alterations in flavonoids. Plant, Cell Environ..

[cit48] Barnes W. P., Flint S. D., Tobler M. A., Ryel R. J. (2016). Diurnal adjustment in ultraviolet sunscreen protection is widespread among higher plants. Oecologia.

[cit49] Ji F., Guo Y., Wang M., Wang C., Wu Z., Wang S., Wang H., Feng X., Zhao G. (2021). New insights into ESIPT mechanism of three sunscreen compounds in solution: A combined experimental and theoretical study. Colloids Surf., B.

[cit50] Duana X., Wub T., Liua T., Yanga H., Dinga X., Chena Y., Mua Y. (2019). Vicenin-2 ameliorates oxidative damage and photoaging via modulation of MAPKs and MMPs signaling in UVB radiation exposed human skin cells. J. Photochem. Photobiol., B.

[cit51] Marcato P. D., Caverzan J., Bergmann B. R., Pinto E. F., Machado D., Silva R. A., Justo G. Z., Ferreira C. V., Durán N. (2011). Nanostructured Polymer and Lipid Carriers for Sunscreen. Biological Effects and Skin Permeation. J. Nanosci. Nanotechnol..

[cit52] Lee S. Y., Lim H. S., Lee N. E., Cho S. O. (2020). Biocompatible UV-absorbing polymer nanoparticles prepared by electron irradiation for application in sunscreen. RSC Adv..

[cit53] Mohamad E. A., Rageh M. M., Darwish M. M. (2022). A sunscreen nanoparticles polymer based on prolonged period of protection. J. Bioact. Compat. Polym..

[cit54] Xu C., Zeng X., Yang Z., Ji H. (2021). Sunscreen Enhancement of Octyl Methoxycinnamate Microcapsules by Using Two Biopolymers as Wall Materials. Polymers.

[cit55] Alvarez-Román R., Barré G., Guy R. H., Fessi H. (2001). Biodegradable polymer nanocapsules containing a sunscreen agent: preparation and photoprotection. Eur. J. Pharm. Biopharm..

[cit56] Lorquin F., Lorquin J., Bruno M. C., Rollet M., Robin M., Giorgio C. D., Piccerelle P. (2021). Lignosulfonate is an efficient SPF booster: Application to eco-friendly sunscreen formulations. Sustainable Chem. Pharm..

[cit57] Zeng Y., He X., Ma Z., Gou Y., Wei Y., Pan S., Tao L. (2023). Coral-friendly sunscreen provides better UV protection than existing options. Cell Rep. Phys. Sci..

[cit58] Englehart J., Lyon B. A., Becker M. D., Wang Y., Abriola L. M., Pennell K. D. (2016). Influence of a polymer sunscreen additive on the transport and retention of titanium dioxide nanoparticles in water-saturated porous media. Environ. Sci.: Nano.

[cit59] Frizzo M. S., Feuser P. E., Berres P. H., Júnior E. R., Campos C. E. M., Costa C., Araújo P. H. H., Sayer C. (2019). Simultaneous encapsulation of zinc oxide and octocrylene in poly (methyl methacrylate-co-styrene) nanoparticles obtained by miniemulsion polymerization for use in sunscreen formulations. Colloids Surf..

[cit60] Li P., Wang S., Zhou S. (2020). Comfortable skin sunscreens based on waterborne cross-linkable polydimethylsiloxane coatings. J. Mater. Chem. C.

[cit61] Virginia Vaudagna M., Aiassa V., Marcotti A., Beti M. F. P., Constantín M. F., Pérez M. F., Zoppi A., Becerra M. C., Silvero C. M. J. (2023). Titanium Dioxide Nanoparticles in sunscreens and skin photo-damage. Development, synthesis and characterization of a novel biocompatible alternative based on their in vitro and in vivo study. J. Photochem. Photobiol..

[cit62] Khan A. U. H., Liu Y., Fang C., Naidu R., Shon H. K., Rogers Z., Dharmarajan R. (2023). A comprehensive physicochemical characterization of zinc oxide nanoparticles extracted from sunscreens and wastewaters. Adv. Environ. Res..

[cit63] Velimirovic M., Wagner S., Koeber R., Hofmann T., Kammer F. V. (2020). Intra-laboratory assessment of a method for the detection of TiO_2_ nanoparticles present in sunscreens based on multi-detector asymmetrical flow field-flow fractionation. NanoImpact.

[cit64] Ilett M., Naveed E., Roncal-Herrero T., Aslam Z., Micklethwaite S., Hondow N. (2023). In situ electron microscopy techniques for nanoparticle dispersion analysis of commercial sunscreen. J. Nanopart. Res..

[cit65] Rodrigues L. R., Jose J. (2020). Exploring the photo protective potential of solid lipid nanoparticle- based sunscreen cream containing Aloe vera. Environ. Sci. Pollut. Res..

[cit66] Lee Y. J., Nam G. W. (2020). Sunscreen Boosting Effect by Solid Lipid Nanoparticles-Loaded Fucoxanthin Formulation. Cosmetics.

[cit67] Widsten P., Tamminen T., Liitiä T. (2020). Natural Sunscreens Based on Nanoparticles of Modified Kraft Lignin (CatLignin). ACS Omega.

[cit68] Wong S. W. Y., Zhou G. J., Leung P. T. Y., Han J., Lee J. S., Kwok K. W. H., Leung K. M. Y. (2020). Sunscreens containing zinc oxide nanoparticles can trigger oxidative stress and toxicity to the marine copepod Tigriopus japonicus. Mar. Pollut. Bull..

[cit69] Niculae G., Badea N., Oprea O. C., Meghea A. (2013). Optimization of lipid nanoparticles composition for sunscreen encapsulation. UPB Sci. Bull. B: Chem. Mater. Sci..

[cit70] Schiavo S., Oliviero M., Philippe A., Manzo S. (2018). Nanoparticles based sunscreens provoke adverse effects on marine microalgae Dunaliella tertiolecta. Environ. Sci.: Nano.

[cit71] Philippe A., Košík J., Welle A., Guigner J. M., Clemens O., Schaumann G. E. (2018). Extraction and characterization methods for titanium dioxide nanoparticles from commercialized sunscreens. Environ. Sci.: Nano.

[cit72] Zhang J., Tian Z., Ji X. X., Zhang F. (2023). Light-colored lignin extraction by ultrafiltration membrane fractionation for lignin nanoparticles preparation as UV-blocking sunscreen. Int. J. Biol. Macromol..

[cit73] Rizzi V., Gubitosa J., Fini P., Nuzzo S., Agostiano A., Cosma P. (2021). Snail slime-based gold nanoparticles: An interesting potential ingredient in cosmetics as an antioxidant, sunscreen, and tyrosinase inhibitor. J. Photochem. Photobiol., B.

[cit74] Vázquez R. N. M., Moisés F. P. P., Rataj V. N., Quijada J. A., Arízaga G. G. C. (2023). Composite with natural ingredients and layered double hydroxide nanoparticles as antioxidant and sunscreen powder material. Mater. Sci. Eng., B.

[cit75] Porrawatkul P., Nuengmatcha P., Kuyyogsuy A., Pimsen R., Rattanaburi P. (2023). Effect of Na and Al doping on ZnO nanoparticles for potential application in sunscreens. J. Photochem. Photobiol., B.

[cit76] Borrás M. C., Sluyter R., Barker P. J., Konstantinov K., Bakand S. (2020). Y_2_O_3_ decorated TiO_2_ nanoparticles: Enhanced UV attenuation and suppressed photocatalytic activity with promise for cosmetic and sunscreen applications. J. Photochem. Photobiol., B.

[cit77] Baek S., Joo S. H., Kumar N., Toborek M. (2017). Antibacterial effect and toxicity pathways of industrial and sunscreen ZnO nanoparticles on Escherichia coli. J. Environ. Chem. Eng..

[cit78] Piccinino D., Capecchi E., Trifero V., Tomaino E., Marconi C., Giudice A. D., Galantini L., Poponi S., Ruggieri A., Saladino R. (2022). Lignin Nanoparticles as Sustainable Photoprotective Carriers for Sunscreen Filters. ACS Omega.

[cit79] Piccinino D., Capecchi E., Delfino I., Crucianelli M., Conte N., Avitabile D., Saladino R. (2021). Green and Scalable Preparation of Colloidal Suspension of Lignin Nanoparticles and Its Application in Eco-friendly Sunscreen Formulations. ACS Omega.

[cit80] Guo L., Wang T., Li Z., Wu S., Xu Y., Yang Z., Li Y., Gu Z., Jiang X. (2023). Melanin-like Nanoparticles Boosted the Photoprotective Efficiency and Stability of Sanshool. Chem. Mater..

[cit81] Fonseca M., Rehman M., Soares R., Fonte P. (2023). The Impact of Flavonoid-Loaded Nanoparticles in the UV Protection and Safety Profile of Topical Sunscreens. Biomolecules.

